# The roles of an urban rooftop garden for the staff of a memory clinic - a qualitative post-occupancy evaluation

**DOI:** 10.3389/fpsyg.2025.1646052

**Published:** 2025-10-27

**Authors:** Nina Oher, Anna Bengtsson, Patrik Grahn

**Affiliations:** Department of People and Society, Faculty of Landscape Architecture, Horticulture and Crop Production Sciences, Swedish University of Agricultural Sciences (SLU), Lomma, Sweden

**Keywords:** roof garden, supportive environment, health promoting, nature, supportive design, salutogenic design, evidence-based design, healthcare staff

## Abstract

The world is experiencing an acute global shortage of healthcare staff, with health and well-being issues, recruitment and retention difficulties. Strategies with potential to improve staff well-being are therefore receiving increasing attention. Contact with nature in the workplace has been shown to help staff recover, reduce stress levels and increase job satisfaction. Additionally, rooftop gardens have become a trend due to the world’s growing urbanization and densification of cities. The aim of the study was therefore to explore the role of an urban rooftop garden for staff at a Memory Clinic, with a specific focus on the physical and health-promoting aspects of the garden. A post-occupancy evaluation (POE) was conducted using qualitative research methodology and focus group interviews, including nine participants (divided into a management – and a staff team) and a total of five interviews. Thematic analysis was used for the transcribed interviews. The evaluation spanned a full year to capture the use, experience and meaning of the rooftop garden in all seasons and possible weather conditions. Three overarching themes and associated sub-themes were identified. The first one, (1) The rooftop garden as a place of Use, promoted both (a) Spontaneous Visits and (b) Organized Activities. The second theme, (2) The rooftop garden as a place to Experience the World Outside, offered (a) Contact with Nature and Surrounding Life and a sense of being (b) Beyond Hospital Walls. The final theme, (3) The rooftop garden as a place of Meaning for Well-Being and Work Life Sustainability, was linked to being either (a) Positive and Rewarding or linked to (b) Temporary wishes and needs for support. Each sub-theme was connected to physical features in the environment, as well as locations (zones) in the garden, which produced results with design significance and potential for practical application and use in planning contexts. The results furthermore show that an outdoor environment such as a rooftop garden can include both salutogenic and pathogenic strategies and therefore be used to both promote health and prevent ill health for staff, that is, provide conditions for optimal support and promotion of health and well-being. Finally, the study highlights urban rooftop gardens as a type of garden with potentially unique, positive and beneficial properties due to its combination of expansive views and urban feel, with calmness, safety, privacy and enhanced seasonal and natural impressions – something that is considered difficult to achieve in an urban hospital garden at ground level.

## 1 Introduction

It is well known through previous research that experiencing a connections with nature in the workplace, such as exposure to natural views, sunlight and/or spending time outdoors can help healthcare staff to recover and reduce their stress levels (Terrapin Bright Green, 2012; Jonveaux et al., 2013; Marcus and Sachs, 2013a; Zadeh et al., 2014; Cordoza et al., 2018; Evans et al., 2019; Copeland, 2021; Nieberler-Walker et al., 2023; Sachs, 2023), and that access to a workplace garden can improve perceived quality of the work environment (Jiang et al., 2018a) as well as increased job satisfaction (O’Hara et al., 2022). This is highly relevant given the global shortage of healthcare professionals (World Economic Forum, 2023; World Health Organization [WHO], 2025a), issues related to staff health and wellbeing (Cordoza et al., 2018; Rudman et al., 2020; Gregory et al., 2023; Martin et al., 2023; Nieberler-Walker et al., 2023; Sachs, 2023), as well as staff recruitment and retention difficulties (Al Zamel et al., 2020; Simonsen and Fleischer, 2024; Sjögren and Parding, 2024).

In light of these current issues related to the health and well-being of healthcare workers, combined with research showing that nature contact promotes recovery and reduced stress levels, the present study draws on theories of restoration, supportive, and health-promoting outdoor environments, which are commonly used in landscape architecture and environmental psychology, such as the attentional restoration theory (Kaplan and Kaplan, 1989; Kaplan, 2001), the stress reduction theory (Ulrich, 1983, 1993), and the calm and connection theory (Grahn et al., 2021; Bengtsson et al., 2025). The study also adopts a Post-Occupancy Evaluation (POE) methodology, as POE’s of outdoor spaces, such as healthcare gardens, have been able to highlight important aspects of the physical environment that are experienced as supportive and health-promoting for their users (Centre for Healthcare Architecture, 2024; Center for Health Design, 2025a), which in turn can lead design recommendations for future healing gardens (Naderi and Shin, 2008; Paraskevopoulou and Kamperi, 2018). POEs furthermore form an essential part of Evidence Based Design (EBD) (Paraskevopoulou and Kamperi, 2018), the emergence and use of which is linked to the increasingly recognized importance of the physical environment in relation to the health and well-being of users (Centre for Healthcare Architecture, 2024). The EBD approach involves incorporating evidence from relevant research, best practices, current knowledge and experience into the design process to create supportive and health-promoting environments for the intended user group (Center for Health Design, 2025b). The completed project and its design should then be evaluated through a POE and the results openly reported for the benefit of current and future projects. The POE thus contributes to higher design quality in both the short and long term (Elf et al., 2017; Brown and Corry, 2020) and is considered an important part of the design process to create “successful” healing outdoor environments (Paraskevopoulou and Kamperi, 2018).

**FIGURE 1:**
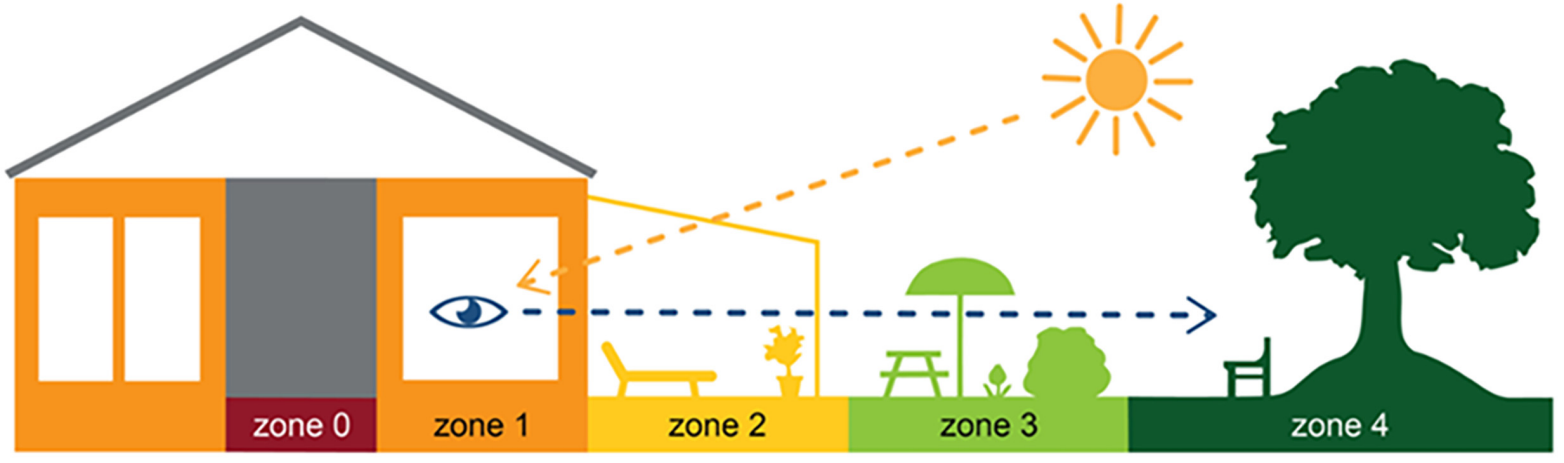
The four zones of contact with the outdoors. Illustration by Anna Bengtsson.

When it comes to health-promoting outdoor environments, a prerequisite for a successful EBD process is to initially collect information (i.e., evidence) about the different areas (zones) of the environment, in terms of their content, qualities and relationships with each other. An evidence-based model called The Four Zones of Contact with the Outdoors, developed by Bengtsson (2015) and used in the present study, identifies four different zones in the physical environment where health-promoting interaction with the outdoors can occur (Bengtsson et al., 2024, 2025). These are: (1) from within a building (e.g., views and daylight from windows), (2) from inside transition zones (e.g., conservatories, greenhouses, balconies, pavilions), (3) in a garden or park (the project site itself) and (4) in the surrounding environment (Figure 1). The purpose of the model is to assist practitioners by highlighting the importance of considering the entire healthcare environment during design processes, all the way from the inside of the building to the outdoors, rather than focusing on isolated areas (Bengtsson, 2015; Bengtsson et al., 2025). The model hence adopts a holistic approach by assuming that the whole is greater than the sum of its parts (Bengtsson, 2015). Creating holistic and informed perspectives for the design of healthcare environments is described as more important in relation to evidence-based design processes than the definition absolute solutions (Centre for Healthcare Architecture, 2024).

Previous research on the needs of healthcare staff in hospital gardens found certain aspects to be particularly important, such as private areas separated from patients and families (Naderi and Shin, 2008; Nejati et al., 2016; Dinu Roman Szabo et al., 2023) with opportunities for both individual privacy and social interaction with colleagues, comfortable, movable and flexible seating enabling use of the garden in different weather conditions, good views all year round, access to nature both visually and physically, aesthetically pleasing outdoor environment, contact with the outside world beyond the work environment, clear thresholds and boundaries, and contrasting features to those inside the hospital, e.g., natural light, quiet natural sounds, abundant greenery, privacy and solitude, as well as soft, colorful and highly textured surfaces.

The implementation of a specific type of outdoor environments, the rooftop garden, has become a rapidly growing and sustainable trend for creating health-promoting green oases in urban environments (Pouya and Demirel, 2017; O’Hara et al., 2022), due to the urbanization and densification of cities (World Health Organization [WHO]., 2025b). It can thus be considered important to evaluate these “new” gardens, to find strategies that can improve staff health and well-being and thereby aid the pressing global issue of recruiting and retaining healthcare professionals. Some POE’s of rooftop gardens have been conducted, for example, in a garden primarily used by patients for physiotherapy, focusing on the successes and weaknesses of the garden design (Davis, 2011), in a built-in rooftop terrace with a view only of the indoor environment (Martin et al., 2021), of rooftop gardens as recreation areas (Pouya, 2019), at a rooftop garden to evaluate the level of suitability of its design based on the healing garden criteria (Sabila et al., 2024), and in hospitals where the evaluation focused on wellness and therapeutic health for patients, staff, relatives and other visitors through the lens of sustainability (Pouya and Demirel, 2017; O’Hara et al., 2022; Starry et al., 2022). However, the authors have not been able to find any previous studies that evaluate the physical aspects of urban rooftop gardens with a specific focus on the use and experience of healthcare staff, as well as the meaning and significance that the garden has for the staff.

The aim of the POE in this study is therefore to explore the role of an urban rooftop garden for staff in a healthcare context, focusing on the physical aspects of the garden. This is done to gain useful knowledge for future design processes where understanding significant physical aspects of a supportive garden, for similar contexts and user groups, is crucial. The following objectives are used to guide the study:

- To examine how the physical design, specific features and zones in the rooftop garden are used and experienced by healthcare staff.
- To identify specific environmental factors that support or hinder restoration and health promotion.
- To highlight the distinctive features and qualities of a rooftop garden, as well as possible advantages and disadvantages compared to ground-based gardens in a healthcare context.

## 2 Materials and methods

This paper describes a post-occupancy evaluation (POE) of a unique urban rooftop garden at a Memory clinic, which was primarily used by its staff. The evaluation of the rooftop garden, designed and built specifically for the Memory Clinic, took place in 2023, approximately 3 years after the clinic opened. Since the clinic’s first year was strongly affected by the COVID-19 pandemic, its impact on the role of the rooftop garden is also included in the study. A qualitative research method consisting of focus group interviews was used, aiming to provide meaningful, in-depth insights into the participants’ experiences, perspectives and behaviors (Gill and Baillie, 2018).

### 2.1 Study design

To explore the role of the urban rooftop garden used by healthcare staff, focus group interviews and photographic documentation of the garden were conducted. The evaluation spanned a full year to capture the use, experience and meaning of the rooftop garden during all seasons and weather conditions of the year. This was significant as Swedish weather offers large seasonal variations, from warm and sunny in summer, to dark and cold in winter.

Two participant groups were created: one with the management team, which was conducted in conjunction with their regular meetings, and a staff group, with the intention of including different professions and work units and thus collecting extensive and comprehensive data.

The Four Zones of Contact with the Outdoors (Bengtsson, 2015; Bengtsson et al., 2025) were used to identify different zones in the garden’s physical environment, for the possibility of making connections between places (including content and qualities), and use, experiences and meaning. The model was furthermore used to note the connection between the different zones, i.e., to consider the impact the zones had on each other in relation to the use, experience and meaning of the rooftop garden.

### 2.2 Setting

In 2020, the newly built Memory Clinic opened in central Malmö, southern Sweden. The clinic is a day center for patient evaluation and treatment with a focus on cognitive diseases such as Alzheimer’s (Region Skåne, n.d.). The clinic also conducts research on memory diseases and dementia care (Starkman Ahlstedt, 2021) and is a world leader in, among other things, research and treatment of Alzheimer’s disease (White, 2021). The Memory Clinic building has furthermore been awarded for its ecological sustainability (White, 2021). The clinic has approximately 140 employees and receives around 450 visitors per week (of which about 250 are patients and 200 are accompanying relatives). The rooftop garden (zone 3) is located on the clinic’s top (i.e., 4th) floor and is approximately 500 square meters in size. The garden is mainly used by staff, but it is also open to patients, relatives and other visitors, who occasionally visit the garden to take a walk or to use it as an outdoor waiting room (with the exception of the COVID-19 pandemic, during which no one except staff accessed the garden).

**FIGURE 2:**
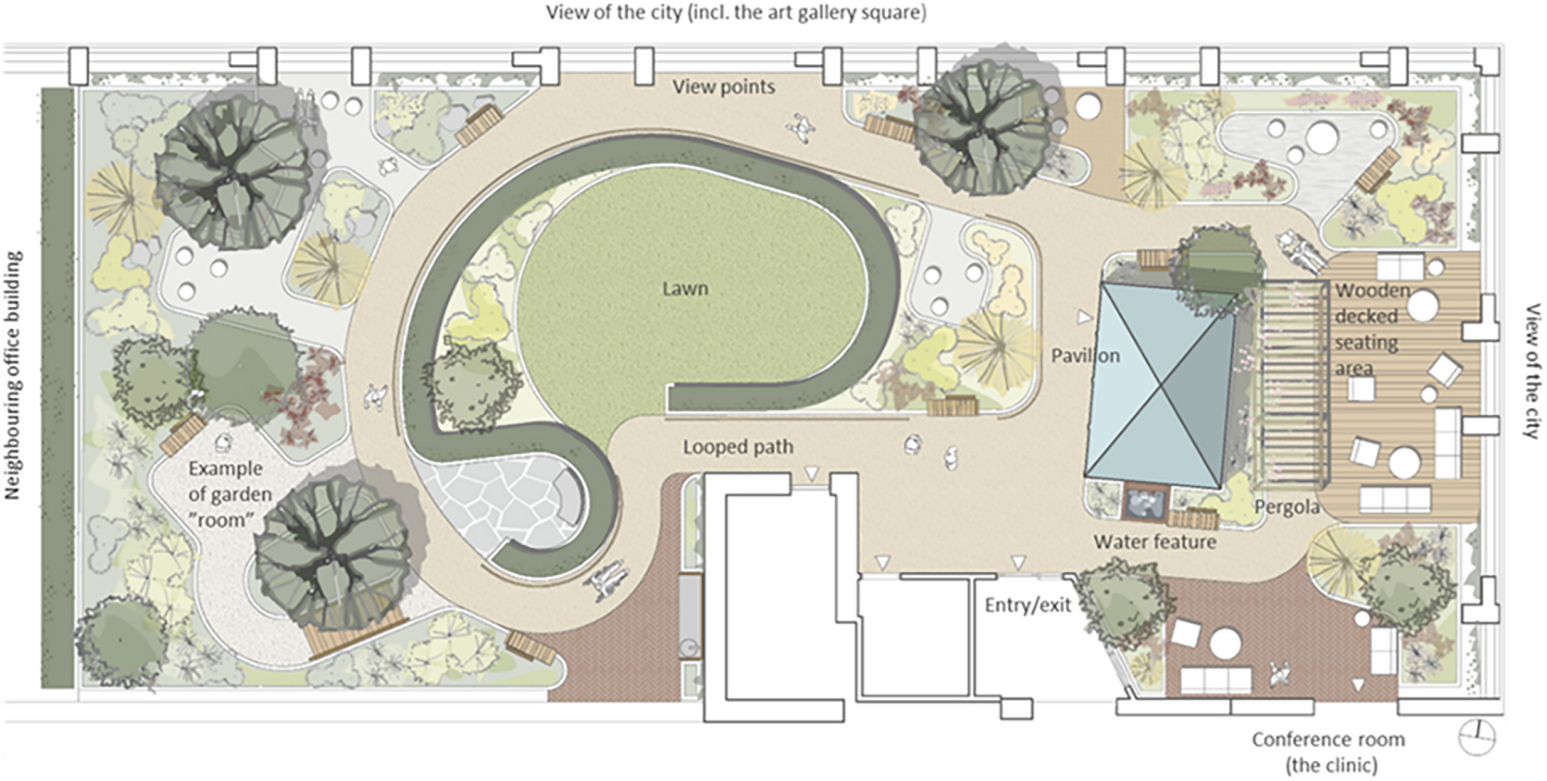
Plan of the rooftop garden. Design and illustration by Markus Magnusson, White Architects. Text added by the authors of this paper.

A wide path wraps around the garden, a so-called “walk & talk” loop. There is a large variety of plants in raised beds, a pergola, a water feature and varying ground materials. In addition, there are open seating areas (space with wooden decking), more secluded and private areas (smaller garden rooms), and a winter-proof (i.e., heated) pavilion (zone 2) (equipped as a meeting room). A central lawn, surrounded by hedges, provides space for physical activities such as exercise and stress management. Viewpoints in the garden offer panoramic views of central Malmö: an art gallery square, a large park and urban development. One of the clinic’s conference rooms is located adjacent to the rooftop garden, with large windows and glass doors that open out to the garden. Although the Memory Clinic has many conference rooms, only this one will be mentioned and discussed in the present study and will therefore simply be referred to as “the conference room.” The illustration below shows the layout of the rooftop garden (Figure 2).

### 2.3 Participants

A total of 9 staff members participated in this study and 5 focus group interviews were conducted: 3 with a management team (3–4 participants) and 2 with a staff team (4–5 participants). The management team was interviewed 3 times instead of 2, due to a project kick-off meeting with the management team where an initial interview was conducted. The participants represented different professions, such as unit manager, senior physician, nurse, counselor and medical secretary. They also worked in different work units within the clinic, such as in the “Mobile Team” that mainly helped patients in their own homes, the team that met and helped patients at the clinic (e.g., in connection with doctor’s visits), the “Memory Health” focusing on physiotherapy and occupational therapy, as well as the Research Unit (Table 1). Of all the participants, 7 of them had worked at the Memory Clinic before moving into the new building (thus having 3 years of experience in the garden) and 2 had been employed since then (with 0.5–1.5 years of experience in the garden).

**TABLE 1:** Demographic characteristics of the focus group interview participants (total number of participants: 9).

Characteristics	Participants
**Gender, *n* (%)**	
–Women	7 (78%)
–Men	2 (22%)
**Profession (work unit), *n* (%)**	
–Unit manager (the mobile team)	1 (11%)
–Unit manager (the clinic)	1 (11%)
–Unit manager (the memory health)	1 (11%)
–Chief physician (the clinic)	1 (11%)
–Nurse (the research unit)	1 (11%)
–Nurse (the mobile team)	1 (11%)
–Nurse (the clinic)	1 (11%)
–Counselor (the mobile team)	1 (11%)
–Medical secretary (the clinic) and safety representative	1 (11%)

#### 2.3.1 Recruitment

The invitation to the management group was sent digitally via a previously established contact within the group. Recruitment of participants to the staff group was also carried out via email, distributed via the respective unit manager. All digital invitations contained a presentation of the study, with an explanation of what participation would entail.

### 2.4 Data collection: focus group interviews

Focus group interviews were chosen to encourage dynamic and interactive group discussions (Morgan, 1996), as well as to achieve more elaborate accounts and collective “sense-making” (Wilkinson, 1998; Wibeck et al., 2007). The interviews were conducted in two rounds; the first was held in the spring to discuss the previous months, i.e., the “cold months,” which covered October to March. The second was held in the autumn and covered the “warm months,” i.e., April to September. The interviews were carried out with a visual connection to the rooftop garden throughout, as they were held either in the garden pavilion or in the adjacent conference room, both of which have glass facades facing the rooftop garden. They were audio-recorded and later transcribed.

Both the first and second author were present during the interviews. To facilitate the discussions and stimulate the participants’ memory, a projected presentation was used during the interviews. This presentation showed a large photo of the rooftop garden (taken from above to see the entire design of the garden) as well as bullet points with interview questions. In qualitative research interviews, stimulus material, such as photos, can be used as a tool to stimulate the conversation by encouraging and reminding the interviewees about the topic in question (Törrönen, 2002; Bell, 2022).

The questions asked during the focus group interviews were based on the study’s research questions. The same interview questions were posed to both the management group and the staff group. The same questions were furthermore asked during the two rounds of interviews, with the exception that interviews in the spring focused on the recently experienced colder months, and the interviews in the autumn aimed to capture the recently experienced warmer months. However, additional questions were asked during the final interview, focusing on the use, experience and significance of the rooftop garden during the COVID-19 period, as the pandemic constituted a significant part of the time the garden had been in use. The following questions were asked during the interviews, with follow-up questions when needed for more in-depth responses.

- Has the garden been USED during the colder/warmer months? (If so, by whom, for what, and when)
- Has the garden offered any EXPERIENCEs during the colder/warmer months? (If so, to whom, what kind, when, from the inside (conference room, pavilion), in contact with the surroundings)
- Do you feel that the garden has had any special SIGNIFICANCE/MEANING during the colder/warmer months? (For you, your colleagues, the clinic operations, the patients and/or next of kin)
- What would you say WORK WELL and LESS WELL about/in the garden, for you and other users?
- What CHANGES/IMPROVEMENTS would you like to see?
- Is there anything special coming up/any PLANS for the garden? (Such as use, events, users, changes, plans)
- Has the garden been used during the PANDEMIC? (If so, by whom, for what, when)
- Has the garden offered any experiences during the PANDEMIC? (For you, your colleagues, the clinic operations, the patients and/or next of kin)
- Do you feel that the garden has had any special significance/meaning during the PANDEMIC? (For you, your colleagues, the clinic operations, the patients and/or next of kin)

#### 2.4.1 Photo documentation

To support the focus group interviews, the rooftop garden was photographed on six different occasions during the POE to capture the garden in different weather conditions and seasons. Aspects such as changes in the garden’s physical appearance and signs of use were of particular interest. All parts of the garden were photographed in a similar way on each occasion. The photographic documentation served as support for the authors during the focus group interviews, as it contributed to a pre-understanding of the garden that helped the authors better follow the interview discussions and more easily ask relevant follow-up questions. The photographs were also used to clarify and exemplify the findings in the present paper and to enhance the overall reading experience.

### 2.5 Data analysis

The analysis was carried out with a focus on the physical environment and aspects related to the physical environment, in line with the aim of the study. The development of themes and sub-themes through thematic analysis, as well as the ethical considerations of the study, are described below.

#### 2.5.1 Development of themes and sub-themes

Thematic analysis was used for the transcribed interviews, a method that facilitates the identification, analysis, and interpretation of patterns (such as themes) within a qualitative data set (Clarke and Braun, 2017). This, mainly inductive (data-driven), process of analyzing data begins by extracting relevant information from the collected data to generate codes, and consequently themes, to help address the study’s research questions. The method is hence suitable for projects that aim to generate themes rather than use pre-determined themes, and that plan to set aside existing theories in favor of new and unconstrained information (Adu, 2021).

Prior to the analysis, the first author transcribed the interviews and then proceeded with the analysis of the collected material. Nvivo 15, a qualitative data analysis program, was used to facilitate and structure the analysis. To increase credibility, the second author then read the transcriptions as well as the analyzed material, i.e., codes, themes and subthemes. The authors discussed the analyzed material until a common consensus was reached. The analysis followed six phases, as suggested by Braun and Clarke (2006, 2012, 2019):

Phase 1: Familiarization with the data

Familiarity with the data was achieved during the transcription of the audio-recorded interviews, which were listened to many times during the process.

Phase 2: Generating initial codes

Appropriate codes were generated for the words, phrases and/or statements that were considered relevant to the purpose of the study.

Phase 3: Generating initial themes

Once all the relevant material had been assigned appropriate codes, the codes were sorted into potential themes. Considerations were made about the relationship between the different codes and how they could be combined into overarching themes, as well as potential main themes and sub-themes.

Phase 4: Reviewing potential themes

All themes, main themes and sub-themes, were re-considered, revised and refined. This was done to ensure that the data within each theme described the same phenomenon and therefore belonged together, and that there was a clear distinction between the different themes.

Phase 5: Defining and naming themes

All the compiled (and coded) data extracts within each theme were read again to see whether or not they appeared to form a coherent pattern. Considerations were also made for each theme, whether it was actually a main theme, sub-theme or even perhaps just a code. This revision and refinement phase led to a rearrangement of the thematic structure by, for example, moving codes from one theme to another, renaming main themes and merging subthemes.

Phase 6: Producing the report

In addition to Braun and Clarke’s suggestion for phase 6, which involved *“the final analysis and write-up of the report”* (2006, p. 93), photos were added to exemplify the study findings and tables were created for each sub-theme, to achieve more design-specific knowledge related to the physical environment.

### 2.6 Ethical considerations

Ethical approval for the study was obtained by the Swedish Ethical Review Authority (reference number 2022-03606-01). The study aimed to investigate the use, experience and significance of the rooftop garden. The focus was not on collecting personal and sensitive information from the staff of the Memory Clinic. However, a conscious effort was made to avoid linking specific statements to a particular profession, as a precaution, to avoid the risk of personal identification. Prior to the interviews, all staff at the Memory Clinic received written information about the study, in which their potential study participation was explained.

All participants were informed that their participation was voluntary and that they could withdraw at any time, without having to give a reason or risking it affecting their work situation in any way. Signed consent forms were collected before the start of the study from the individuals who agreed to participate.

## 3 Results

**FIGURE 3:**
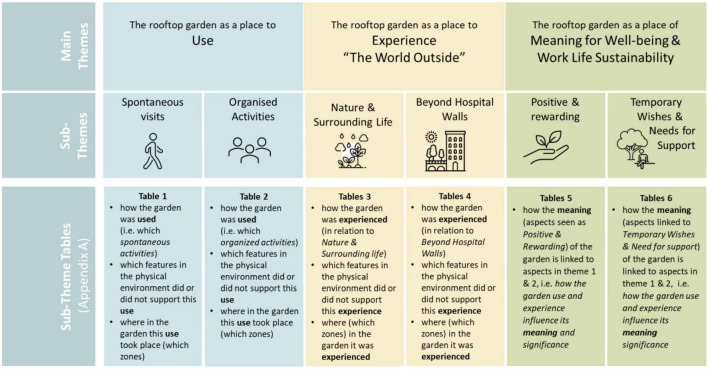
Main themes and sub-themes of the result, as well as table content for each sub-theme (see Supplementary Appendix A).

The results indicate that the rooftop garden can be and function differently, that is, play different roles, depending on aspects such as users, needs, situation, time spent in the garden as well as time of year. Based on the analysis of the focus group interviews, three main themes were identified: (1) The garden as a place for Use, (2) The garden as a place to Experience the World Outside, and (3) The garden as a place of Meaning for Well-being and Work life Sustainability. Each main theme was followed by two subthemes each, resulting in a total of six subthemes. All themes are presented in Figure 3. Additionally, each sub-theme is presented in individual tables, see Supplementary Appendix A.

### 3.1 The garden as a place to use

The rooftop garden provided the staff at the Memory Clinic with access to an outdoor space to use during their workday. The garden was used in a variety of ways, ranging from Spontaneous Visits to Organized Activities. These different ways of using the garden were furthermore connected to different features and places (zones) in the environment (Supplementary Tables 1, 2 in Appendix A).

#### 3.1.1 Spontaneous visit (a)

**FIGURE 4:**
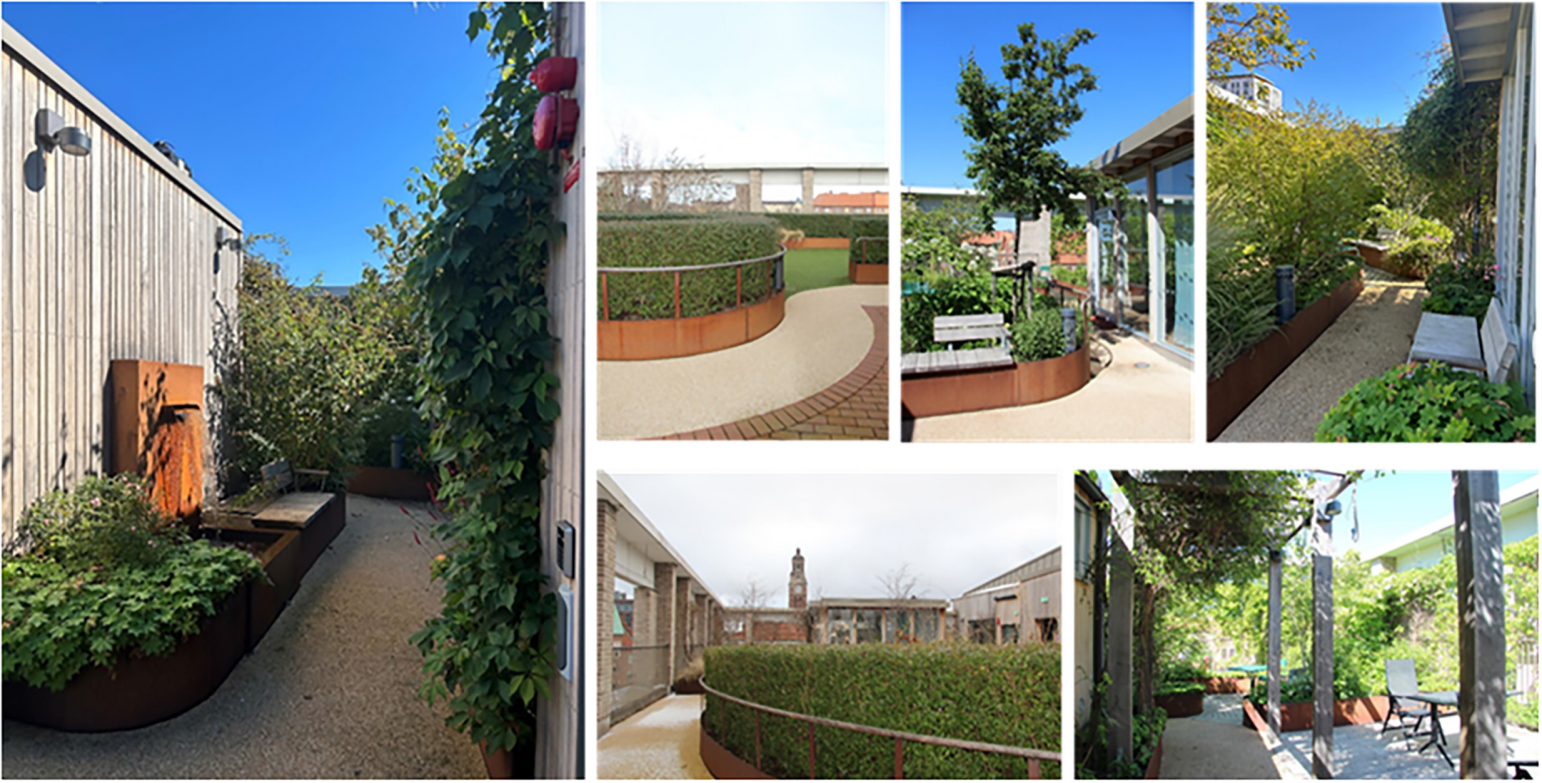
View from the rooftop garden of the surrounding city. Source: Authors.

**FIGURE 5:**
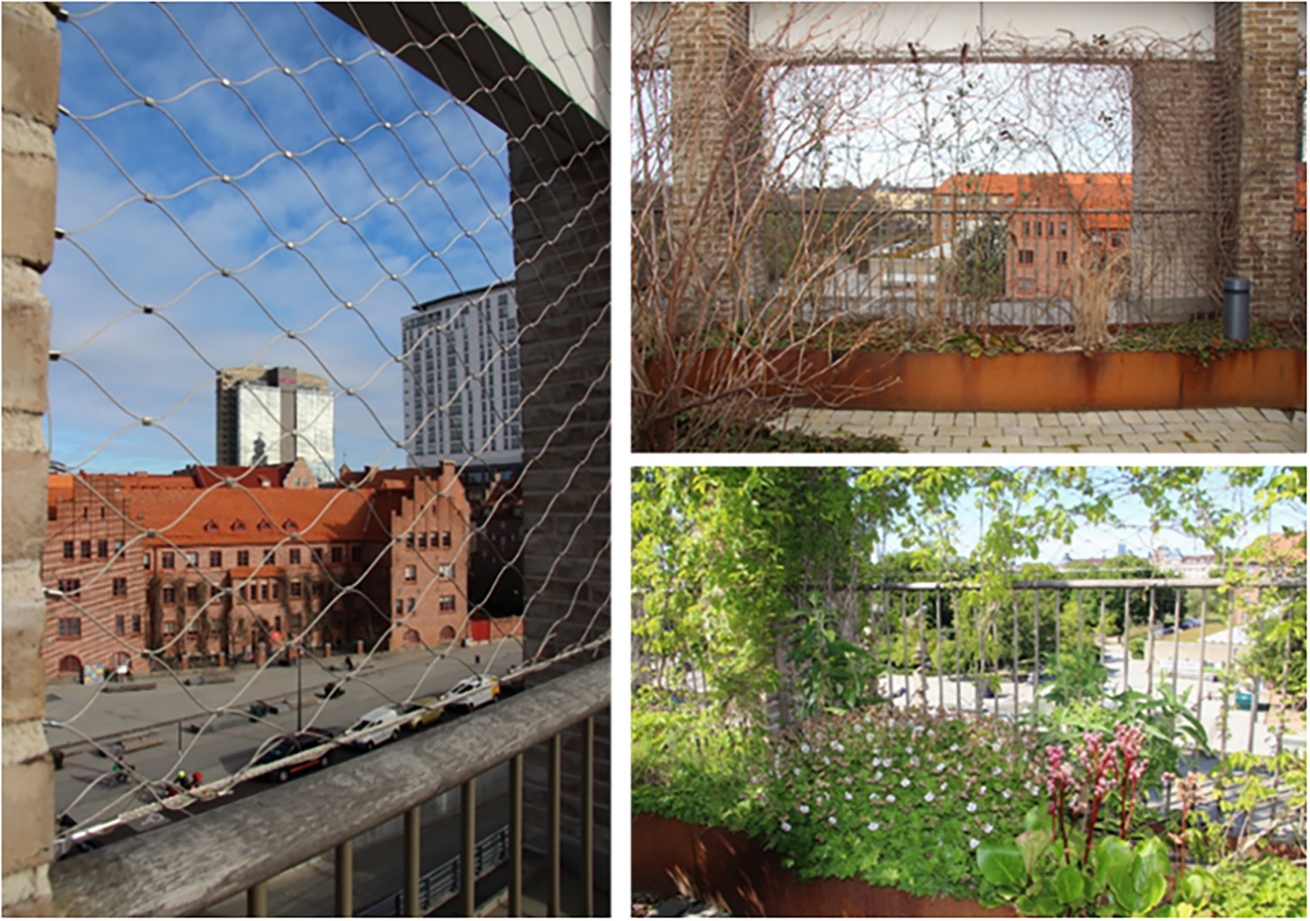
View from the garden to the surrounding city. Source: Authors.

**FIGURE 6:**
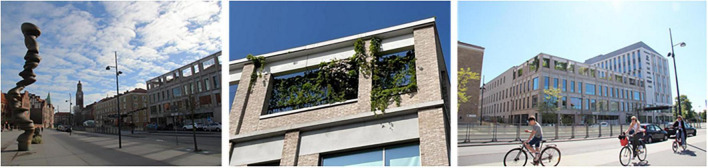
The rooftop garden seen from the outside, in its urban location. Source: Authors.

Spontaneous visits consisted of shorter breaks (“micro breaks”) where staff went out to the garden for various reasons. The garden was, for example, used for taking walks along the looped path of the garden (Figure 4), and for getting daylight and fresh air. Another reason for going out to the garden was to look at the vegetation, to note changes and developments in the garden such as discovering new buds in springtime. The staff also enjoyed standing by the railing in the garden and looking out over the city and down toward the art gallery square (seen in Figures 5, 6). This was done and appreciated all year round. Experiencing the view of the city was one of the main reasons for going out into the garden, according to the staff. “You go outside to get that micro break, but also to see what’s going on in the garden and to get a view of Malmö.” Micro breaks were furthermore achieved on the way out to, and back from, meetings in the conference room and the pavilion: “being able to get out just the 10 m, I think that feels nice actually.”

**FIGURE 7:**
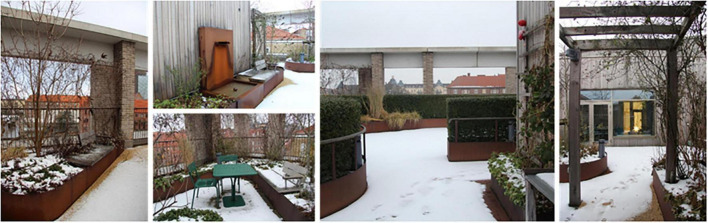
The roof garden in winter, when the view of the city becomes the main attraction. Source: Authors.

**FIGURE 8:**
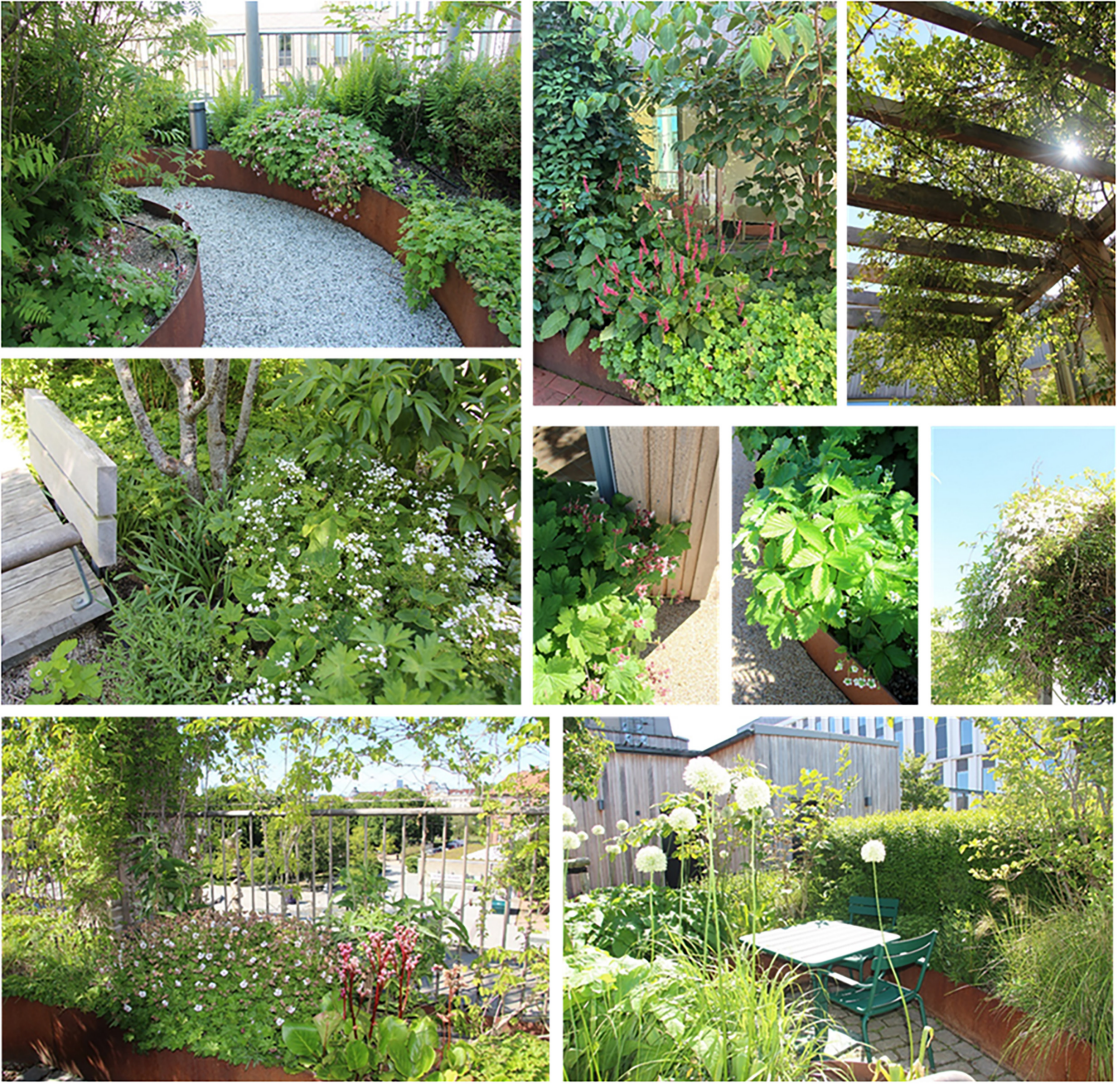
Variety of plants and flowers (species richness). Source: Authors.

**FIGURE 9:**
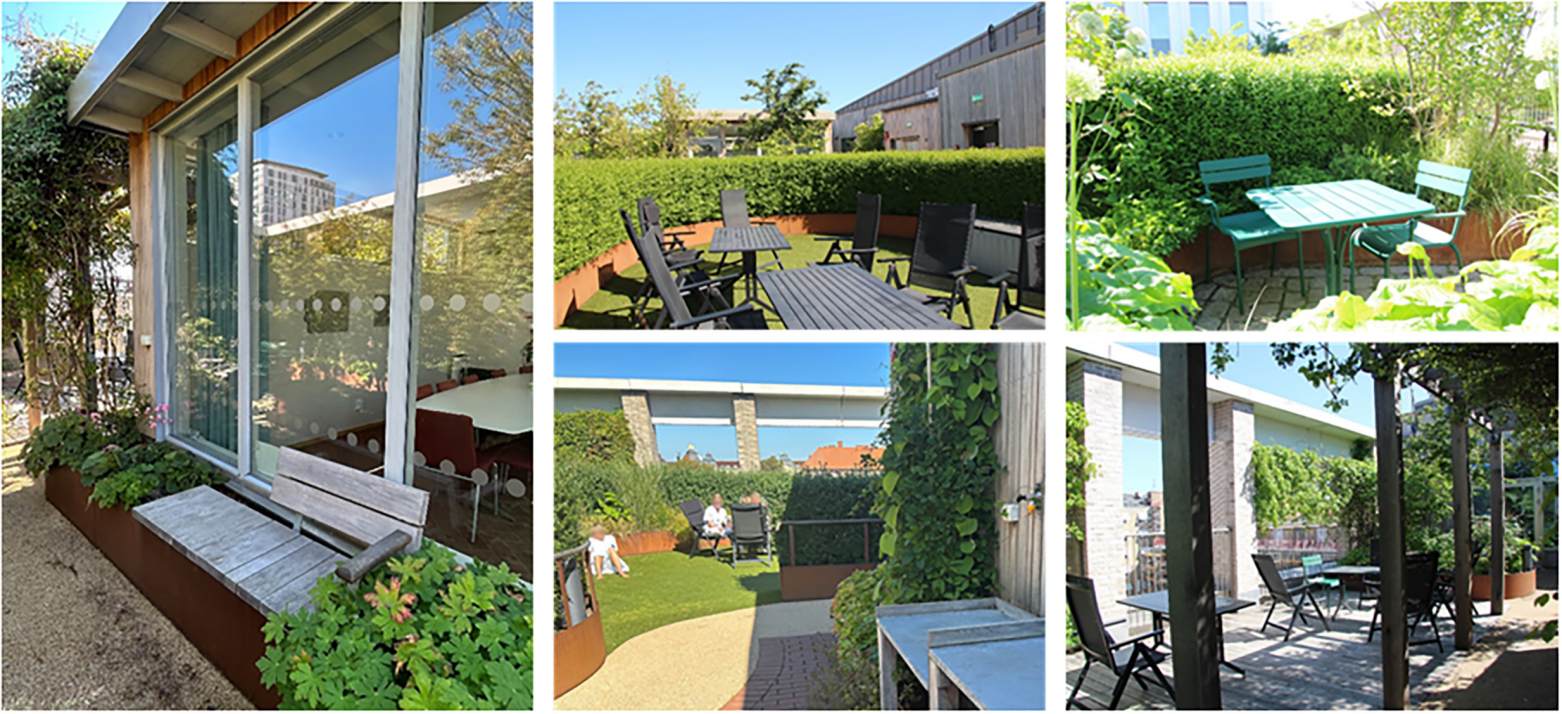
A variety of seating: some fixed (left), some easily moved, some in open areas and some in more private ones, some in the sun, some with more shade. Also visible: the pavilion (far left), the lawn (center), the deck (far right, below), and a smaller garden “room” (upper right corner). Source: Authors.

Occasionally even quick and spontaneous workouts could be carried out. Staff described how they saw a young colleague, still dressed in white, doing about 10 pull-ups out in the garden by the pergola (seen in Figures 4, 7–9), before going back in again. *“There probably aren’t many workplaces where you can just go outside and do some pull-ups,”* they reflected.

#### 3.1.2 Organized activity (b)

When it was possible for staff to stay in the garden for a longer period than just a short break, it was used to eat lunch or have a coffee, either in the garden when warm and sunny or inside the pavilion on colder days. Flexible and easily moved garden furniture made it possible to sit in different configurations (alone, in small or larger groups) and in various places (open spaces or more private, in the sun or shade) in the garden (Figure 9), which was appreciated.

**FIGURE 10:**
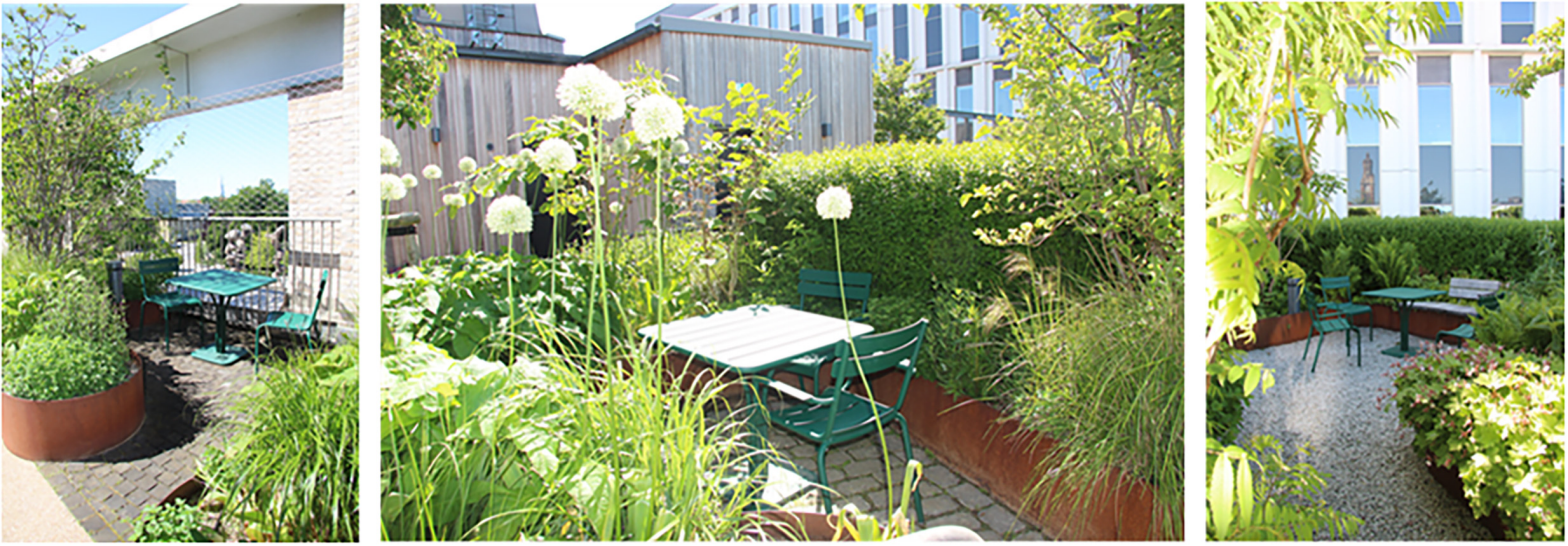
Garden rooms. Source: Authors.

The garden was also used for work-related tasks such as administrative work, although some staff found it difficult to read and use laptop screens outdoors. Activities such as reading, staff meetings (often held in the pavilion) or “walk n’ talk” conversations between colleagues in the garden were perceived as more possible by all. The smaller garden rooms off the main path were used for small group conversations, individual work, or to be alone for a while (Figure 10). *“There are these little rooms, these little secluded spaces that are very good to sit in and read and concentrate.”* There were, however, concerns related to the extent of privacy offered by the garden rooms. Although they were perceived as more private due to the surrounding vegetation, there was still a risk that conversations held there could be heard in adjacent parts of the garden, which made it difficult to discuss sensitive work-related issues when other people were around.

**FIGURE 11:**
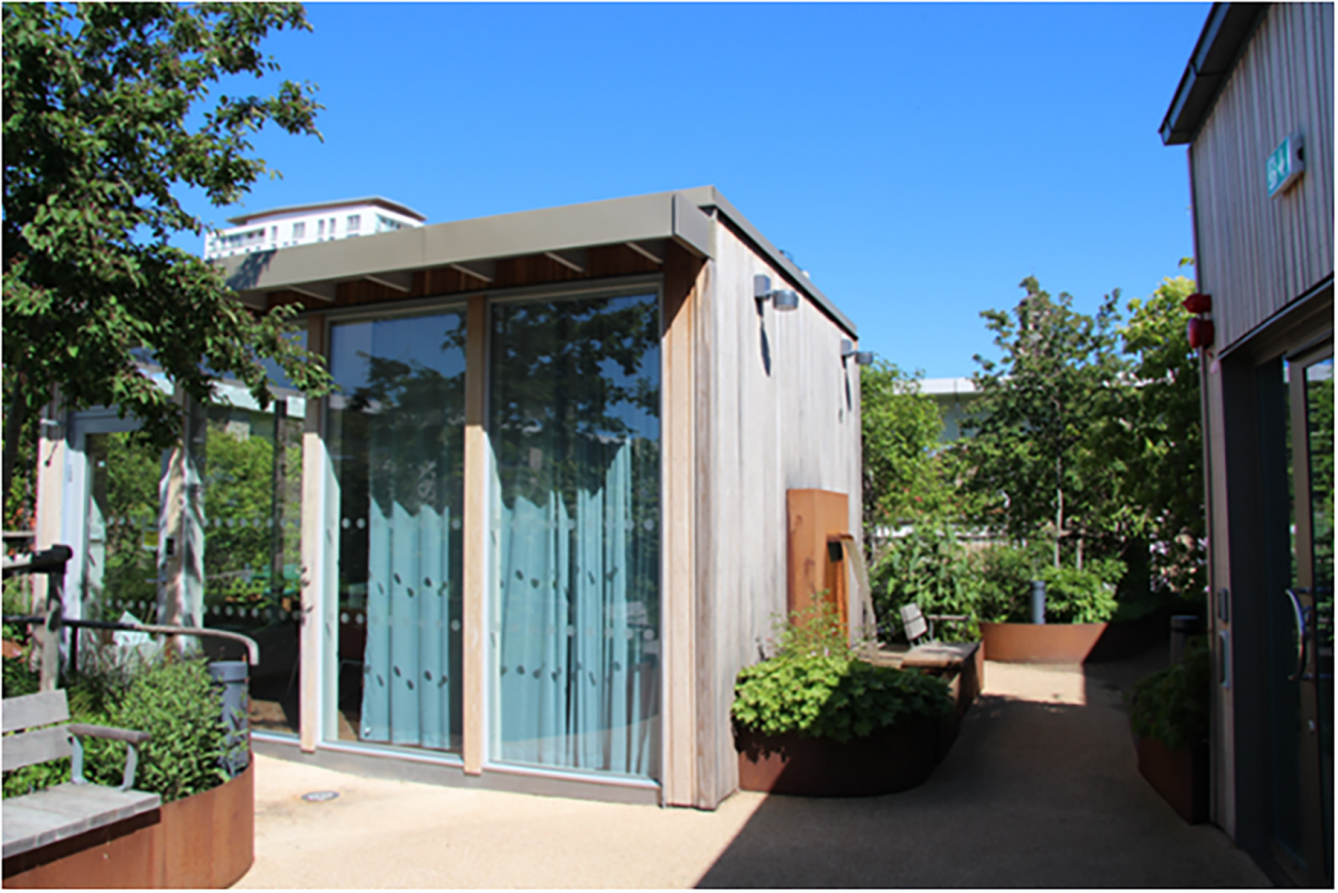
The pavilion. Source: Authors.

Additionally, the garden provided a space for preventive health care activities, such as exercise (walking or using the outdoor gym equipment), and mindfulness (usually on the round lawn in the middle of the garden, seen in Figure 9), which seemed particularly appreciated, and possible (due to the smaller number of staff remaining at the clinic), during the COVID-19 pandemic. *“Staff went up [to the garden] with this exercise app because as it turned out you needed to channel quite a lot of emotions [anger and stress].”* After the pandemic, when the large proportion of staff who had been temporarily working and at other healthcare facilities came back to the clinic, the conditions for outdoor exercise changed. Apart from the pavilion (seen in Figures 9, 11), it was not possible to book any part of the garden, which meant that staff who wanted to exercise without others seeing them chose to do so indoors in private rooms instead.

**FIGURE 12:**
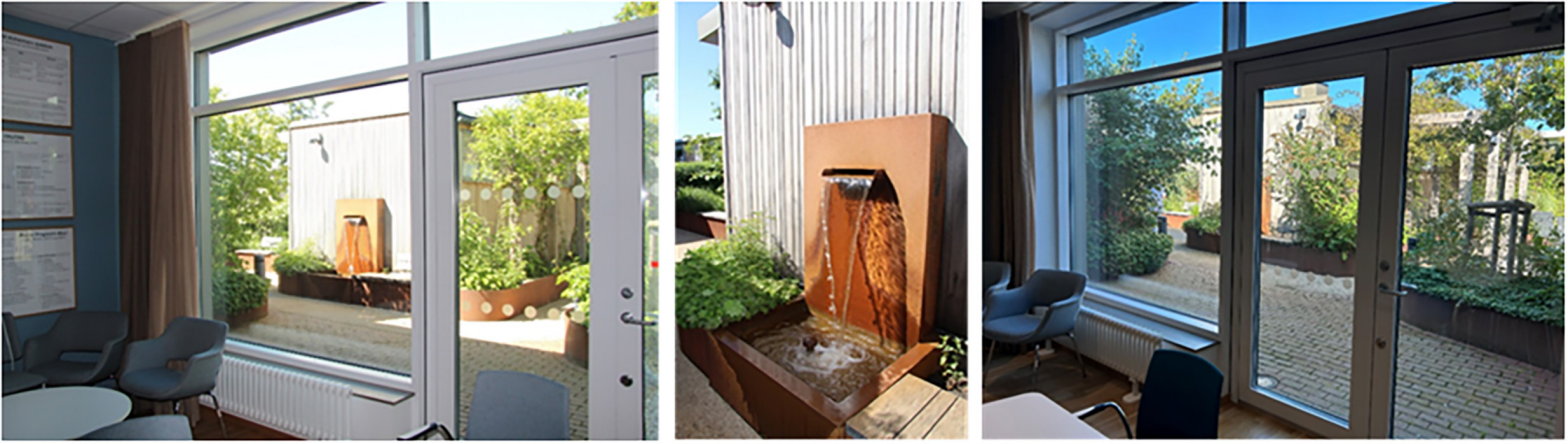
View from the conference room to the garden. Source: Authors.

The garden furthermore provided an outdoor space for staff to gather for joint activities and various celebrations (weather permitting). During the colder months, the conference room overlooking the garden became a popular place to gather instead (seen in Figures 7, 12). When it got dark outside (which happens relatively early in the afternoons during Swedish winters), it was still nice to look out due to the outdoor lights in the garden. It created a nice atmosphere and made the conference room a nice place to gather: “it’s lit up, so it creates a pretty beautiful picture outside. This lighting here is actually inviting, it’s like a painting.”

### 3.2 The garden as a place to experience the world outside

The rooftop garden was experienced as a connection between the clinic and the outside world, through the contact and proximity to nature and surrounding life (the city) that it provided. Additionally, the garden was a place that, despite belonging to the clinic, was experienced as being situated outside of the healthcare facility. This contributed to a feeling of being somewhere else, where one could escape for a while. The theme is thus divided into (a) Contact with Nature and Surrounding Life, and (b) Beyond Hospital Walls. These different ways of experiencing the garden were furthermore connected to different features and places (zones) in the environment (Supplementary Tables 3, 4 in Appendix A).

#### 3.2.1 Contact with nature and surrounding life (a)

**FIGURE 13:**
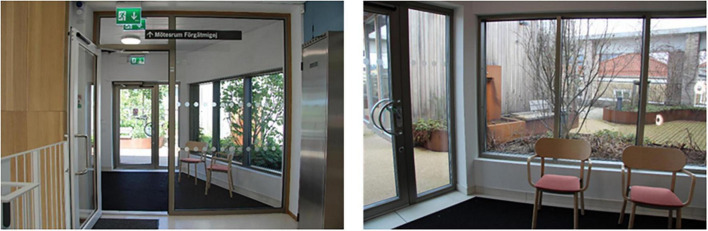
View of the garden from inside the clinic corridor. Source: Authors.

A connection and closeness to nature was experienced, both physically in the garden and visually from the pavilion, through the windows in the conference room and clinic corridors (those on the same floor as the garden). The visual contact meant a connection to nature even when it was too cold or wet to go outside (examples in Figures 12, 13). The rooftop garden also made it possible for staff to follow the seasons (including weather conditions during the day) and notice changes in nature (e.g., leaves changing color in autumn). During the warmer months, the plants grew quickly and abundantly, making the garden green and lush, something the staff believed was a result of an existing microclimate. In winter, when snow came, it stayed longer on the rooftop garden than down at ground level. *“Here [in the conference room] you can also see that it is winter when you look out, you don’t get that feeling when you look out from the canteen to the courtyard [at ground level]”* said one participant (for rooftop garden in winter see Figure 7). Experiencing this contact and closeness during working hours is unusual for healthcare professionals, staff pointed out. *“If you compare it to some hospitals that I have worked in, you are deep inside some kind of bunker and have no connections at all to the outside world. So it’s cozy with the conference room [which have a visual connection to the garden].”*

A connection to nature was also achieved through visiting animals, such as insects and birds, who were attracted by the garden’s flowers, plants and water feature.

**FIGURE 14:**
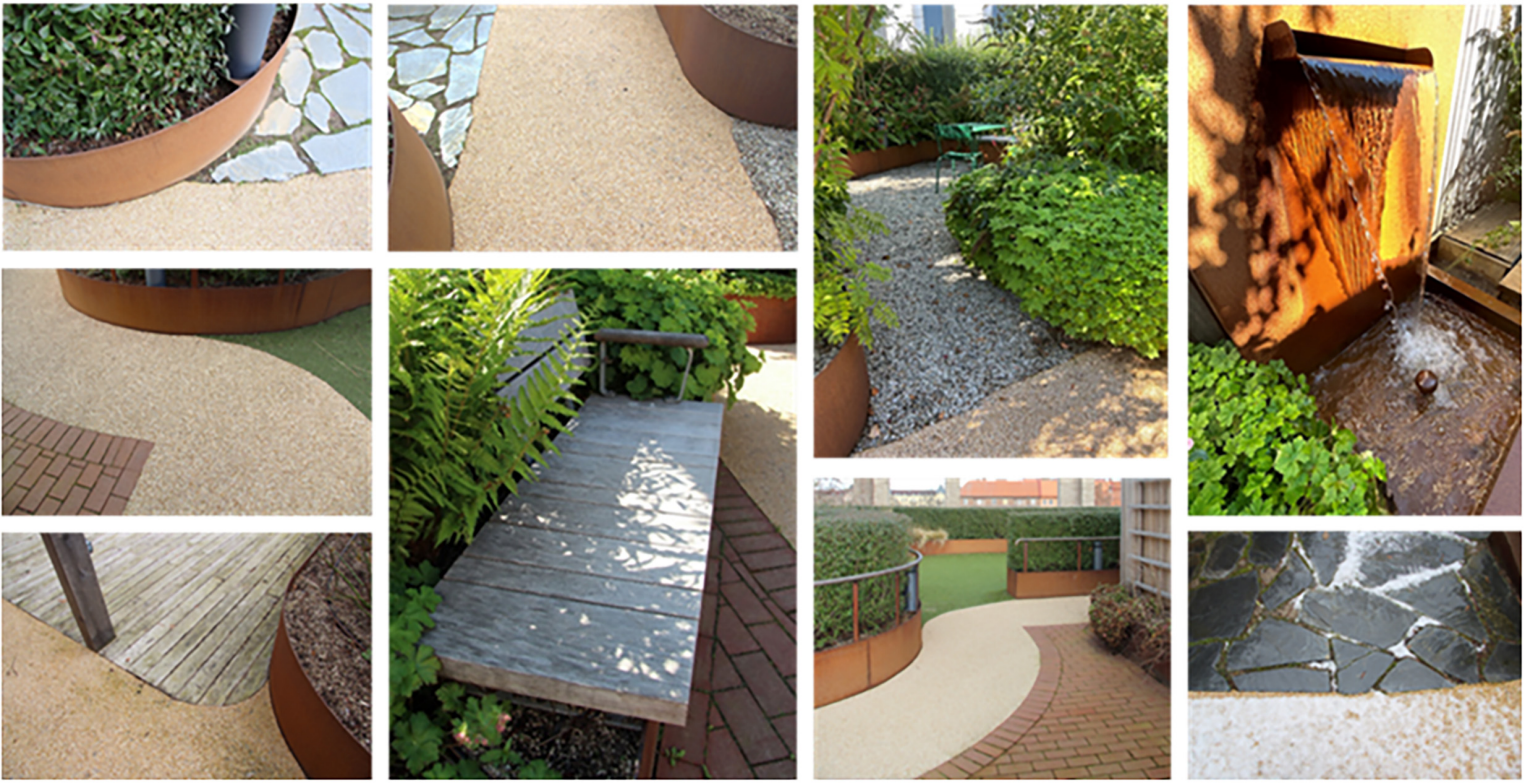
Variety of materials, patterns, colors and structures. Source: Authors.

Natural elements such as fresh air, light, sunshine, and the garden’s diversity of plants and flowers contributed to sensory experiences for the staff (seen in Figure 8). The water feature was a particularly popular detail in the garden, described as both pleasant to look at (visible also from inside the conference room) and pleasant to listen to [seen in Figures (4, 12, 14)], as the rippling sound could be heard in a large part of the garden, as well as from inside the pavilion. However, it was considered a bit of a nuisance that the water feature had to be operated by a member of staff to keep it running, and that its function was somewhat affected by the wind. The ground material of the looped path around the garden also provided sensory experiences (made of rubber granules), and was described as both attractive and “setting the mood”; a sensation that was palpable as soon as you stepped onto it (seen in Figures 4, 9, 14) and contributed to a feeling of wanting to go barefoot: “You sometimes feel like “Oh, I could take my shoes off [here]”.” Since the garden offered a variety of sensory stimuli, some of the staff had been interested in starting mindfulness walks. These would include simple signs at various points in the garden, suggesting that people stop, look, smell, touch or listen to different elements in the garden. Due to the sensory stimulation the garden offered it was furthermore seen as a good environment for certain types of work tasks: “There is a lot of scent, a lot of color, and many shapes to fill the brain within the middle of the day. I think it is wonderful and that it is easier to perform certain tasks up here, which have to do with creativity and creation, new thinking. Then the rooftop garden can be a good environment to sit in. The mind becomes a little freer up here with a view of the rooftops, with air and light and vegetation.” (See Figures 8, 14 for variety of colors, shapes etc.).

The attractive, open and unobstructed view was an important part of the garden’s appeal and a contributing factor to the overall experience of it. The staff appreciated the central location in the bustling city combined with being able to be high above in a quiet and private “bubble.” The contrast between the proximity to nature that the garden offered and the urban environment was experienced as special and unique. *“It can be nice to stand and look out to see what people are doing, like cycling to work and school. Sometimes you can just stand there and look and wonder where they are going. Feeling that there is a pulse outside, but that right here it is still calm.”* During the colder months, when the garden was dormant, the view of the city was described as its most attractive feature (seen in Figures 7, 5) and the view became the main reason why the staff went out into the garden. Impressions were thus taken from outside rather than from within the garden itself during the colder months of the year. Some participants, however, wished for more covering “green walls” facing the city (i.e., more climbing greenery on the mesh) for increased sense of safety, with possible peepholes to look out through, while other participants thought it would be better to keep the view as open as it was (Figures 5–9).

The staff appreciated how accessible the rooftop garden felt to them and appreciated the ability to go out quickly and easily, just for a few minutes, without having to change out of work clothes or put on a jacket. However, there was a difference in how accessible the garden felt, and thus how often it was used, depending on where in the clinic and on which floor the staff had their workplace. Staff working on the top floor, i.e., the same floor as the rooftop garden, experienced a visual connection to the garden (through windows), which reminded them of its existence (Figure 13). *“Good yes. Especially when you go to get coffee from the coffee machine, it’s so easy, just walk a few more steps [to get to the garden].”* Staff who worked on the lower floors, on the other hand, tended to easily forget about the garden and explained that they have to make a conscious decision to go up to the garden for it to happen. *“You have to take the time to go up, so you don’t just go out and have your coffee here [in the garden], but you have to make a decision “Should we go up?” And that makes it more difficult.”* The physical distances to the garden required a certain effort that thus affected the use of the garden, according to staff.

#### 3.2.2 Beyond hospital walls (b)

Spending time in the garden was experienced differently compared to spending time inside the clinic. Visually, the garden was of course different from the indoor environment and made it very clear to the staff that they were now somewhere else. The aesthetically pleasing design of the garden contributed to a feeling of wanting to be there. It felt inviting and was experienced as a coherent whole (and as particularly pleasant during the warmer months of the year), according to the staff. The garden offered a calm, quiet and peaceful environment. The sounds of the city formed a distant background noise, which was not perceived as disturbing to the staff. “It is still very quiet for being in the middle of the city, that’s because we are high up, I guess.” Even at lunchtime in the summer, when the garden was at its most crowded, it was still possible to find quiet corners. Privacy and seclusion was thus experienced in the garden, both in relation to other people in the garden, thanks to the smaller garden rooms (Figure 10) or the pavilion, that almost always made it possible to be alone (or in smaller groups), but also in relation to the city outside: “[You are outside but] it is still private, you’re kind of on your own. When you stand on the street, you are one of many. Here you’re on your own.”

From a social perspective, the garden was also experienced as different and more flexible than the indoor environment. The lack of dedicated seating meant that everyone sat wherever they wanted (such as on the outdoor furniture by the wooden deck, directly on the lawn or in the more private garden rooms surrounded by plants and shrubs), which led to staff meeting more often across unit boundaries.

### 3.3 The garden as a place of meaning for well-being and work life sustainability

In addition to the use and experience of the garden described in theme 1 and 2 above, the results also revealed aspects linked to its meaning and significance. These aspects were generally described as either: (a) Positive and rewarding or linked to (b) Temporary wishes and needs for support. Positive and rewarding (a) represent aspects in the garden that generally seem to have a positive and rewarding impact on the staff. Temporary wishes and needs (b), on the other hand, represent aspects in the garden that correspond to the needs and wishes that may arise on certain occasions, such as in particularly stressful situations. Common to these aspects was their combined impact on staff well-being and job satisfaction, and thus also on work life sustainability.

Supplementary Tables 5, 6 in Appendix A indicates a relationship between the three themes by illustrating how the use of the garden (theme 1) and the experience of the garden (theme 2) influenced aspects linked to the meaning and significance of the garden (theme 3). Theme 3 captures what is inherently less tangible and therefore cannot be as easily linked to specific physical features and places in the rooftop garden. However, it highlights what is seen to have a more direct impact on the health and well-being of staff, i.e., what has meaning and significance, such as for example feelings of renewed energy, increased job satisfaction and restoration.

#### 3.3.1 Positive and rewarding (a)

Visits to the garden were seen as meaningful, positive and rewarding, as they led to new energy for the staff, generated by, among other things, the breaks, fresh air and beautiful environment that the garden provided. *“It’s incredibly beautiful, I was really happy when I went out and saw it. It kind of gave me energy. Just going out to get some fresh air, but I got energy [from the garden] at the same time.”* Even a short visit, like a quick walk in the garden or a look toward the art gallery square below, was felt energizing, and having access to sunshine, fresh air and vegetation was considered both health-promoting and stress-preventing, with a positive impact on their job satisfaction. *“It’s so incredible to have something like this [the garden] I think about when I worked in a hospital ward, you were barely outside all day. It doesn’t feel like you’re so confined here. I think it’s beneficial for our job satisfaction.”* Staff pointed out that their enjoyment of the garden could also have a positive impact on patients: *“I think it has to do with preventive healthcare and well-being, so that if we feel good, the patients get good care, so it rubs off.”*

Having free access to the private outdoor environment of the rooftop garden contributed to a meaningful sense of freedom for the staff. “It’s that feeling of freedom, a breathing space, to get a break, that you don’t have to go out into the city but you can go out here. It’s some kind of freedom, and calmness.” The garden furthermore seemed to have a positive impact on the staff even when it was not being used or experienced. One staff member described that just knowing that the lovely garden with the nice view was there, was meaningful. Another said: “I think the significance [of having a garden] also has a mental aspect - knowing that I have the opportunity to go outside. There is a place of retreat in my mind, so to speak.”

The garden was described as a source of pride for the entire clinic; it was a given place to show off to visitors, including potential new employees in connection with recruitment and job interviews, as well as attracting great curiosity from outside, such as study visits from architectural firms who wanted to take a look and be inspired. Having access to the garden, especially on warm, sunny days, felt luxurious and contributed to a sense of pride and privilege for the staff. *“We, the sun worshipers. It’s nice to lie down especially at the beginning of summer. Then we usually lie on the grass after lunch and just. You long for the sun. You dream that you are on the beach or somewhere else. It’s actually quite luxurious”* (seen in Figure 9). Staff reflected about the uniqueness of having a rooftop garden with easy access to the outdoors during the working day: *“Imagine the time you change jobs and end up somewhere else and don’t have [a rooftop garden]*… *Then you’ll understand how much joy you’ve had from it.”* Even seeing the garden from the outside, which was possible in all seasons, inspired feelings of pride, according to the staff: *“You can see it [the rooftop garden] from the outside too, because I often go out to the park and that’s what distinguishes this house from the other houses, you see that “There’s our garden””* (Figure 6).

#### 3.3.2 Temporary wishes and needs for support (b)

Certain meaningful aspects of the garden were connected to moments that triggered temporary wishes or specific needs for support. An example was the view of the garden from the conference room, described as important to staff as it provided an opportunity for positive distraction. This was particularly appreciated, and needed, in connection with tough and stressful meetings, staff explained. The view of the garden gave them something to look at and talk about, such as the water feature, the weather and the visiting birds in the garden (Figure 12).

During the colder and grayer months of the year, when it was primarily the view of the city that was appreciated in relation to the garden (as mentioned previously), the garden still had the potential to instill hope, the staff described. *“Even though it [the rooftop garden] feels a little dreary and a little sad, it still gives a sense of calm. It kind of rests in the winter. Then at the end of the winter you see that things start to happen a little bit [e.g., buds on plants start to appear]. You start to feel a sense of hope, so to speak.”*

The garden provided both a sense of retreat and refuge, that is, it was seen as a place to rest and regain energy, as well as a place to escape when feeling sad or angry. The latter was especially true during the pandemic, according to the staff: *“It’s calming with all the flowers and stuff and you can actually “hide” in a corner. For me it was like getting away from everything that was going on in there [at the clinic, during the pandemic]. You’re not as visible there [in the garden], you can be alone for a while and collect yourself a bit.”* During this stressful time the garden was seen as helpful and supportive, contributing feelings of hope and normality, as well as offering good opportunities for recovery *“It [the garden] perhaps had the same meaning, in that it provided a different environment and that it was calm, but I needed it [the garden] much more at that time [during the pandemic].”* The garden was described as an oasis, a place to escape to, a refuge where one can breathe and rest. During the pandemic, it was seen as helpful and supportive, contributing feelings of hope and normality.

Even after tough staff meetings, or between longer patient visits, staff found the garden to be a refuge where they could clear their heads, “blow off steam” and breathe before going back inside. The analysis revealed that “being able to breathe” in the garden was mentioned in both a figurative and metaphorical sense. This expression was partly about access to fresh air, which according to staff was considered particularly important since the clinic had no openable windows. In addition, “being able to breathe” was about the possibility of getting away, having a break, a moment to gather oneself and a chance to recover.

## 4 Discussion

This study aimed to explore the roles of a rooftop garden for healthcare staff, focusing on the physical aspects of the outdoor environment. The goal was to contribute with useful and design-related knowledge for future projects, related to the specific combination of urban rooftop gardens, healthcare context and staff use – relevant in an era of urbanization, environmental issues and acute shortage of healthcare workers worldwide. By identifying features in the environment (that either support or do not support the use and experience of the garden) in combination with its location (and zone) in the garden (Supplementary Tables 1–4 in Appendix A), this study contributes clear and practical information that can be used in a design context. The results indicate that the roles of the garden for healthcare staff can be divided into three overarching themes: (1) The garden as a place to Use, (2) The garden as a place to Experience the Outside World, and (3) The garden as a place of Meaning for Well-Being and Work-Life Sustainability. This part of the paper discusses important findings in relation to these three themes, including the relationship between the garden zones, which corresponds to the study’s first and second objective: (i) To examine how the physical design, specific features and zones in the rooftop garden are used and experienced by healthcare staff, and (ii) To identify specific environmental factors that support or hinder restoration and health promotion. The tables in Supplementary Appendix A also correspond to the first and second objective of the study, as they link garden use and experience to physical features and layout, and illustrate their impact on staff health and recovery. Furthermore, results are discussed in relation to what distinguishes a rooftop garden from a ground garden, which constitutes the third objective of the study: (iii) To highlight the distinctive features and qualities of a rooftop garden, as well as possible advantages and disadvantages compared to ground-based gardens in a healthcare context. Finally, the strengths and limitations of the study are discussed, as well as suggestions for future studies.

### 4.1 The rooftop garden as a place to use

The use of a rooftop garden is strongly influenced by weather and seasons, and thus its geographical location. The variation in the Swedish climate, with both cold and dark, as well as warm and bright months, meant that both weather protection (e.g., for shade) in the rooftop garden, and more sheltered and partially (or fully) indoor environments were a prerequisite for year-round use and experience. This was especially true in relation to organized activities (sub-theme 1b), where the pavilion and conference room offered sheltered spaces but with a strong visual connection to the garden. The need for and importance of a heated greenhouse, pavilion or similar structure to enable health-promoting contact with nature and year-round use has been mentioned previously (Söderback et al., 2004; Davis, 2011; Pálsdóttir, 2014; Bengtsson et al., 2024; Oher et al., 2024). Similarly, but in relation to spontaneous visits (sub-theme 1a), the view appeared to play a crucial role in the use of the garden throughout the year, especially in relation to the colder months when the garden itself was not perceived as attractive. During these months, it was primarily the view of the city, rather than the garden itself, that encouraged staff to go outside and thus access outdoor breaks, fresh air and daylight – aspects of the outdoor environment that have previously been shown to provide positive health and well-being benefits and increased job satisfaction (Terrapin Bright Green, 2012; Jonveaux et al., 2013; Marcus and Sachs, 2013a; Zadeh et al., 2014; Nejati et al., 2016; Jiang et al., 2018b; Evans et al., 2019; O’Hara et al., 2022; Nieberler-Walker et al., 2023). This suggests that a pavilion (or similar garden structure) as well as an interesting and attractive view both constitute significant aspects in connection the use of health-promoting gardens for staff, especially in climates similar to Sweden.

Easy access and proximity can be seen as a prerequisite for connection to a garden, nature and surrounding life (sub-theme 2a). This, in combination with the garden’s visibility from the inside of the hospital, is mentioned in many studies as important design considerations with benefits for both user orientation and time efficiency (Davis, 2011; Nejati et al., 2016; Pouya and Demirel, 2017; Cordoza et al., 2018; Jiang et al., 2018a; Sachs, 2023). However, the present study found that the use of the garden connected to visibility, not only to facilitate orientation but as a reminder to the staff of its existence and as encouragement for them to visit and use the garden. The staff that could see the garden daily from inside the building described how they used the garden to a greater and more spontaneously extent, whereas the staff without visual contact to the garden admitted to forgetting it was there. The visibility between the rooftop garden in relation to staff workstations, break rooms, corridors, the coffee machine or anywhere else staff visit regularly should therefore be carefully considered to optimize garden use.

### 4.2 The garden as a place to experience the world outside

The staff, a professional group often with experiences of working in bunker-like work environments, particularly appreciated and valued being in contact with the “outside world,” i.e., experiencing seasonal conditions and changes outside the hospital’s walls, during their working day. The current study found that experiencing cyclical properties of nature provided staff with a sense of hope and normality, particularly during the colder and darker months, as well as during the extremely stressful COVID-19 pandemic. Existing research suggests that the living, growing and ever-changing qualities of nature reflect its ability to adapt to the stresses of survival, leading to feelings of safety, hope and life (Stigsdotter and Grahn, 2002; Heerwagen, 2009; Kellert and Calabrese, 2015). This again justifies the need for rooftop gardens that encourage year-round experiences, that is, also during the colder months of the year, for example through appropriate plant selection, attractive views and heated pavilions or greenhouses.

Being beyond the hospital walls (sub-theme 2b) emerged as an important experience of the rooftop garden and shows similarities to the feeling of “being away,” described by the ART theory (Kaplan, 2001). To get away or withdraw from what is experienced as draining, to an environment that is *“physically or conceptually different from one’s usual environment”* (Kaplan and Kaplan, 1989), has been highlighted as an important aspect of restorative environments (Marcus and Sachs, 2013b) for healthcare staff by providing “…*a sense of normality, a different perspective, a break from focusing on the trauma and illness*…” (Reeve et al., 2017, p. 54). In the present study, the difference between the clinic and garden was reinforced by sensory experiences (ground material, water feature, plants etc.), the open and far-reaching views, as well as the noticeable contrast between extreme closeness to nature in the garden and the urban environment surrounding it. Opportunities for privacy and seclusion emerged as an important part of the garden, with the sense of enclosure providing a reassuring sense of safety for staff. This is consistent with the quality Shelter, described as *“Where the visitor is offered a secluded safe place while maintaining contact with the outside world*… *usually emerges in smaller, somewhat enclosed spaces, preferably in the protection of vegetation”* (Stoltz and Grahn, 2021, p. 26). Exposure to this quality has furthermore been associated with lower stress levels (Grahn and Stigsdotter, 2010). In contrast to the quality just mentioned, open views were also one of the most valued features of the garden. Previous studies have both concluded that open and enjoyable views reduce feelings of confinement (as open views contrasts positively with common hospital interiors) (Naderi and Shin, 2008; Jiang et al., 2018b), and that healthcare gardens should provide feelings of physical enclosure and safety (Marcus and Sachs, 2013b). The fact that seemingly opposite characteristics, such as openness and enclosure, were valued in the rooftop garden suggests that a supportive environment should offer a spectrum of functions in the garden to meet a variety of users’ needs and desires, as has been previously described (Stoltz and Grahn, 2021; Bengtsson et al., 2024).

### 4.3 The garden as a place of meaning for well-being and work-life sustainability (3)

**FIGURE 15:**
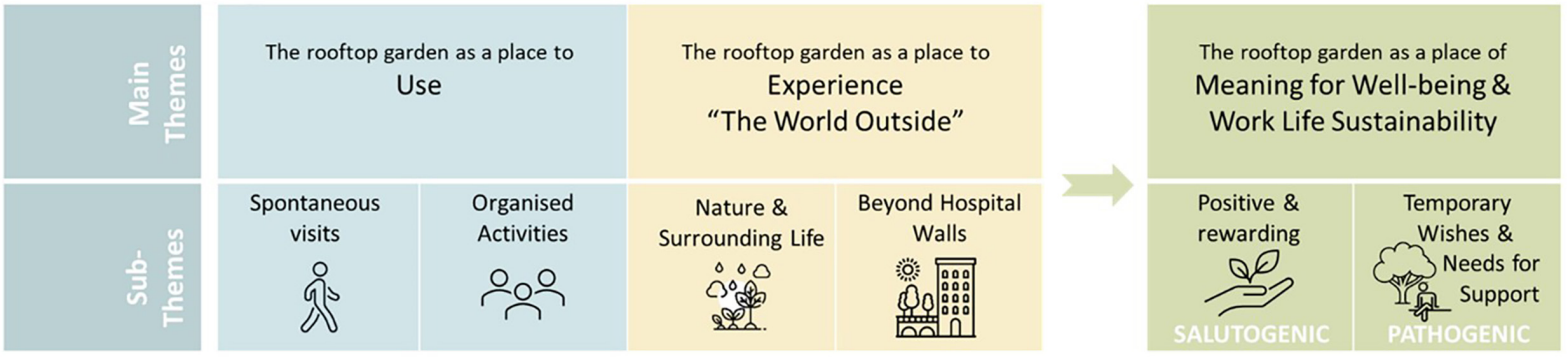
Salutogenic and pathogenic qualities connected to theme (3) the rooftop garden as a place of meaning for well-being and work life sustainability.

The sub-themes for the garden as a place of Meaning for Well-Being and Work-Life Sustainability (theme 3): Positive and rewarding (3a) and Temporary wants and needs for support (3b), indicate that the garden has roles that are clearly linked to salutogenic versus pathogenic perspectives (Figure 15).

Positive and rewarding (3a) can be seen to have a salutogenic effect on staff as the garden was experienced as a place of retreat, associated with feelings of freedom and pride, as well as with health-promoting, stress-preventing properties that increased job satisfaction. Positive and rewarding can thus be described as promoting health and well-being by proactively creating, strengthening and improving physical, mental and social well-being (Antonovsky, 1987; Becker et al., 2010; Bengtsson et al., 2018). However, in relation to Temporary wants and needs for support (3b), the garden offered support that was particularly meaningful in stressful situations, by providing a refuge with positive distractions, possibilities for recovery, a sense of normality and hope, as well as a place to breathe (figuratively and literally). This is in line with pathogenesis which describes factors that cause disease, ill health and stress, as well as how to avoid, cure or eliminate them (Antonovsky, 1987; Becker et al., 2010; Bengtsson et al., 2018). The results thus show that an outdoor environment such as a rooftop garden can include both salutogenic and pathogenic strategies and therefore be used to both promote health and prevent ill health for staff, that is, provide conditions for optimal support and promotion of health and well-being, as previously pointed out by Bengtsson et al. (2018). These results highlighting work-life sustainability are considered relevant, especially from a contemporary perspective, since many healthcare professionals today experience poor health, and as we are experiencing an acute shortage of healthcare professionals globally (Cordoza et al., 2018; Scheffler and Arnold, 2019; Rudman et al., 2020; World Health Organization [WHO], 2020, 2025a; Buchan et al., 2022; Ulrich et al., 2022; Gregory et al., 2023; Michaeli et al., 2024).

Salutogenesis and pathogenesis can further be linked to the concepts of retreat and refuge (as exemplified in the text above), as these are not considered synonymous with each other in this context. They both connect to the “garden-as-escape” experience, described by Naderi and Shin (2008) as a connection with the outside world, beyond the work environment, to achieve a desired sense of escape and distance from work-related stress and fatigue, with opportunities for positive physical and mental distractions. However, a retreat, which in everyday language refers to a place that offers rest and relaxation in a calm, pleasant and private environment (Cambridge University Press Dictionary, 2025a; Merriam-Webster Dictionary, 2025a; Oxford University Press Dictionary, 2005a), could arguably have salutogenic benefits by supporting health and well-being (by providing, for example, renewed energy, increased job satisfaction and sense of pride). A refuge, on the other hand, provides a respite and safety from a stressful and/or uncomfortable situations (Cambridge University Press Dictionary, 2025b; Merriam-Webster Dictionary, 2025b; Oxford University Press Dictionary, 2005b), allowing for relief and a chance to recover (by providing, for example, positive distractions, restoration and a sense of normality and hope), and therefore can be seen to have pathogenic properties.

The need to “breathe” in the rooftop garden was mentioned repeatedly in connection with the pandemic and proved to have a double meaning: partly as being able to breathe (figuratively) as the staff were allowed to take off their protective face masks in the garden and thus were able to breathe, see and talk to their colleagues more easily, and partly as an expression of the (metaphorical) feeling of being able to relax, pause and have a chance to recover. In a similar way “fresh air” has previously been highlighted as one of the most beneficial aspects of going outdoors (Marcus and Barnes, 1995; Nejati et al., 2016; Sachs, 2017, 2019) with the suggestion that “fresh air” could have more than one meaning by also symbolizing change, a break, or a sense of escape. The importance of natural outdoor spaces for recovery, especially in relation to stressful situations like a pandemic, is well documented (Lottrup et al., 2013; Nejati et al., 2016; Cordoza et al., 2018; Copeland, 2021; Gola et al., 2021; Iqbal and Abubakar, 2022; Dinu Roman Szabo et al., 2023; Nieberler-Walker et al., 2023) and further confirmed by this study, as the garden functioned as a much-needed refuge for the staff during this time. This justifies the need for supportive outdoor environments in healthcare contexts, where stress and crisis are commonly occurring. Furthermore, as mentioned by Fernemark et al. (2022), the COVID-19 pandemic is probably not the last crisis to impact healthcare organizations and therefore this study constitutes an important addition to the discourse on improving the work environment for healthcare staff now, thereby contributing to improved well-being and a stronger workforce better prepared for the future (Gola et al., 2021; Iqbal and Abubakar, 2022; Sierakowska and Doroszkiewicz, 2022; Gregory et al., 2023; Sachs, 2023).

### 4.4 The relationship between the zones of the rooftop garden

**FIGURE 16:**
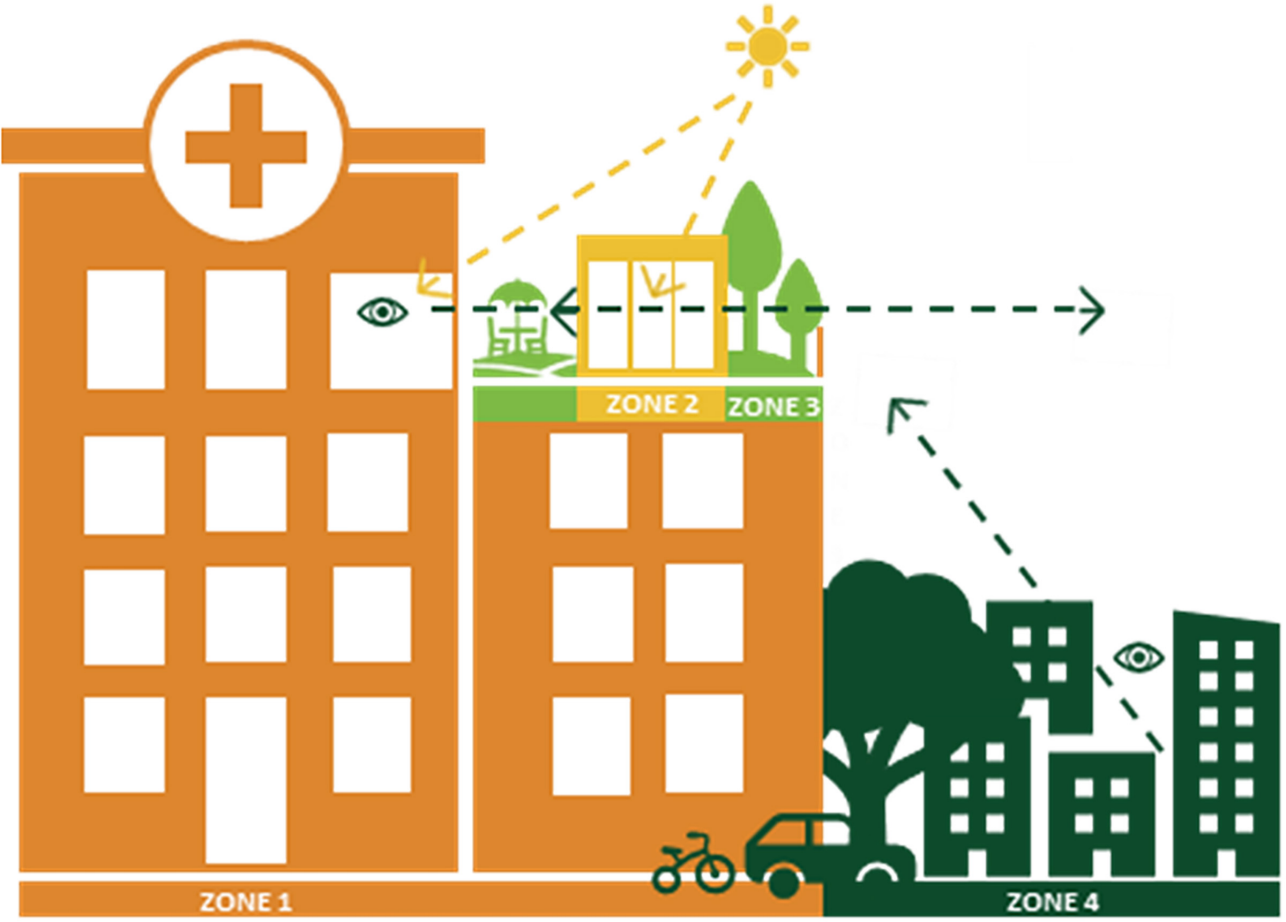
The different zones of the rooftop garden, with arrows indicating their contact with each other [developed by Nina Oher from the original model by Bengtsson (2015)].

There was a noticeable connection and relationship between different places, or zones, in the rooftop garden, in accordance with the model. The four zones of contact with the outdoors (Bengtsson, 2015). Figure 16 illustrates the different zones of the rooftop garden, with arrows indicating the connection between the zones.

The visual connection from Zone 1 (the clinic corridors and conference room) to the garden (zone 3) were significant for staff for various reasons; it allowed them to follow the weather and season (i.e., be in contact with the “outside world”), be reminded of the garden’s presence and encouraged to go outside, as well as to enjoy the garden views, daylight and positive distractions.

The pavilion constitutes Zone 2 of the rooftop garden, a transitional zone with extensive visual garden contact, due to the glass facade on two sides of the structure (as well as smaller windows on the third wall). The use of the pavilion meant visits to the garden that might not have otherwise taken place, especially during the colder months of the year. Although the distance from the clinic was short, the walk in between was considered a pleasant break and an opportunity to get some fresh air. This justifies the placement of a Zone 2 structure further out in the garden rather than right next to the healthcare building.

Zone 3 consists of the garden itself, which is influenced by (and itself influences) all its surrounding zones: the clinic (zone 1) and the pavilion (zone 2) whose structures (including materials, location, size, etc.) are a strong part of the overall experience of the outdoor environment, as well as the surrounding environment (zone 4) which contributes views, openness and space. This is consistent with the argument that a hospital garden never exists in isolation (Pangrazio, 2013) but stands in a spatial relationship with the medical buildings (Jiang et al., 2018b) and its surroundings (Bengtsson et al., 2024). An understanding of this relationship can be seen as a prerequisite for a successful outcome and must be considered and planned for already in the design phase.

Zone 4, the surroundings, proved significant for the staff and defining for the rooftop garden. Its open and far-reaching views gave staff the reason they needed to visit the garden during the colder months, when the garden itself was dormant, and provided them with impressions from the world outside as well as positive distractions. Contact between the garden and its surroundings furthermore existed in the opposite direction, as the garden and its greenery could be seen from the street. This was appreciated by the staff as it contributed to a sense of pride when looking up at the rooftop garden on their way to work, or when they heard that others had seen “their” garden from street level.

In addition to confirming the relevance of the zone model, this study has further developed the model to show the 4 zones of a rooftop garden. This developed and type-specific model further highlights the relationship between zone 3 and zone 4, also in the “opposite” direction, which is indicated by an arrow starting in zone 4 and pointing toward zone 3. This is due to the importance of being able to see the rooftop garden (zone 3) from the city outside (zone 4), expressed by the staff in the study. It is likely that green elements in the cityscape, such as visible rooftop gardens, can be appreciated also by the city’s passing residents.

### 4.5 What distinguishes a rooftop garden from a ground level garden?

The rooftop garden of the Memory Clinic, an inner-city healthcare environment with significant space constraints that limit ground-level gardens, is a current example of how design can support the development of healing gardens in a dense urban environment. However, the result sparked reflections on whether the garden would have been used and experienced similarly, and held the same meaning for the staff, if it had been a ground-based garden. Rooftop gardens are seen as a good alternative for introducing more green spaces in dense areas where space is a rare commodity, and as a creative way to recover the benefits of lost green spaces in cities (Pouya and Demirel, 2017; O’Hara et al., 2022). Are rooftop gardens therefore seen as a good “plan B,” that is, are rooftop gardens solely better than no garden at all, or are there advantages linked to garden use and experience that are unique to rooftop gardens? The attractive, open and unobstructed view was an important part of the garden’s appeal and a contributing factor to the overall experience of it. The staff enjoyed the central location of the bustling city, while at the same time being able to stand high up in their quiet and private “bubble.” The contrast between the calmness, privacy and proximity to nature that the garden offered, with the urban environment and the impressions and distractions that it provided, was experienced as special and unique, which led to enjoyment and a sense of pride. The results of this study hence indicate that the location of the garden, i.e., being high up instead of at ground level, clearly influenced the overall and positive experience of the environment.

### 4.6 Strengths and limitations

The use of qualitative focus group interviews in combination with photographic documentation provided methodological triangulation and is thus seen to increase the credibility of this study. Spreading the data collection across all seasons is furthermore considered to have strengthened the ecological validity of the results. However, despite the achieved variation among the participants (female and male participants, different professions, tasks, positions and work unit affiliations), the small sample size (*n* = 9) could have been larger had the recruitment process generated more participants, which would have increased the generalizability of the study. As a result, the findings should be used with some caution.

Recruitment was carried out via email, on repeated occasions, and distributed via management. In retrospect, it might have been more successful to invite the staff directly, for example in connection with a clinic meeting with a large part of the staff present, to further clarify the focus and significance of the study, and to emphasize that everyone’s opinions and experiences were of interest. Although a larger number of participants would have been preferable, it is believed that larger focus groups, and thus more staff engaged at the same time, would have negatively impacted on the clinic’s daily operations. It proved to be a challenge to find interview times that suited the recruited participants due to their busy schedules. The high workload in healthcare can make it difficult to recruit study participants, as previously mentioned by Fernemark et al. (2022). Therefore, it is considered more ideal in this context to plan for several smaller focus groups, rather than larger groups with more participants in each.

Since the interviews were conducted on repeated occasions but with the same focus groups, it gave the participants a chance to reflect on the use, experience and meaning of the rooftop garden between sessions, and to bring their thoughts and recent experiences to the next interview. It also meant an opportunity for management to ask their employees questions that they themselves had been unsure about before the next session. This opportunity to think, reflect, discuss and ask colleagues between interviews can to some extent be seen as compensation for the small sample size and thus partly increase the generalizability of the study. In addition, empirical studies have shown that smaller groups provide greater opportunities for participation, as well as more focused and in-depth conversations, than larger groups (Wilkerson, 1996). Wibeck et al. (2007) argue that a relatively small group is necessary to achieve an atmosphere that supports a range of perspectives, which is desirable in a focus group situation. The interviews generated many engaged, relevant and informative discussions, where a significant amount of interesting data was obtained. By talking to each other, the staff themselves gained insights and came up with ideas and solutions, such as improvements to their garden, which was exciting for both the researchers and the staff to experience. The displayed photograph of the garden was furthermore found to stimulate conversations during the interviews and help participants remember different ways in which the rooftop garden had been used and experienced, which is in line with existing literature (Törrönen, 2002; Bell, 2022).

Finally, dividing managers and staff into two different focus groups proved to work well. Although the management group generously shared their experiences during the interviews, most of the group had a general tendency to explain how things worked and why. The staff group, on the other hand, was to a greater extent able to discuss what worked well and what worked less well in the garden and allowed themselves to express what was missing, as well as how they wished the garden could be used instead. The difference in how the questions were answered in the two groups can be seen as understandable due to the different roles that the participants held. Nevertheless, this is important to highlight in relation to grouping of focus group interviewees. This experience indicates that separating management and staff groups can provide insight into possible differences in perspective, as well as provide opportunities for more unfiltered and exploratory answers.

The timing of the evaluation, 3 years after completion, was strongly influenced by the COVID-19 pandemic, which struck shortly after the clinic opened. The evaluation began after the pandemic, when daily operations had returned to normal and when the garden had been used for what was perceived as a sufficient period. It was important to allow the feeling of novelty to subside before the evaluation, as this can affect how the environment is experienced (Center for Health Design, 2025a; Bulman, 2022). Although the evaluation was carried out later than initially planned, timeframes mentioned in the literature were met. While previous studies suggest that POE assessments of buildings should be conducted somewhere between 6 months and 1 year (Shepley, 2011; Center for Health Design, 2025a) or within 4–24 months of construction (Vischer, 2002), the timeframe mentioned for gardens is within 3–5 years (Naderi and Shin, 2008), or simply after the gardens have “been in use for a reasonable period” of time (Heath and Gifford, 2001, p. 24). Furthermore, it was important for the current study to understand the role of the rooftop garden for staff in all types of weather conditions and seasons, which is why the POE was conducted over a full year, which is different from some previous studies where assessments were conducted during the summer “to capture favorable weather for outdoor breaks” (Cordoza et al., 2018, p. 509).

### 4.7 Future research

As the study involved a relatively small number of participants, it is believed that more studies with a similar focus should be conducted, with the potential to increase generalizability of the current findings and contribute to knowledge of how the physical design of rooftop gardens can best meet the needs of healthcare staff. The tables in Supplementary Appendix A could be further developed to serve as knowledge base in the design discussion when planning outdoor environments in healthcare. Another way to increase knowledge in this area could be to focus on (i) different groups of healthcare professionals, (ii) rooftop gardens used for a wider range of activities by both staff, patients and visitors, or (iii) rooftop gardens that are part of the daily operations of the healthcare facility (e.g., for therapeutic activities for patients). Finally, comparisons and investigations of the advantages and disadvantages of urban rooftop gardens compared to ground-based gardens are suggested for future studies.

## 5 Conclusion

This study has shown that the significance and meaning of a rooftop garden for the healthcare staff who occupy it is influenced by (i) the uses it offers and (ii) the experiences it enables. The significance and importance of the garden could further be linked to salutogenic and pathogenic aspects, indicating that rooftop gardens can be used to both promote health and prevent ill health for the staff, that is, provide conditions for optimal support and promotion of health and well-being. The rooftop garden stood out as particularly important for the healthcare staff during the stressful time of the COVID-19 pandemic, where the need to “get away” from the clinic to an environment that was experienced as visually and conceptually different, was of extreme importance. Additionally, this article highlights what distinguishes a rooftop garden from a round-based garden in a health-care context, which is, among other things, its lofty location that allows for interesting contrasts and diverse views, where people, vehicles, life and movement can be seen from a distance in a place that feels peaceful, green and private. The ability to look out over the city from a distance is also of great importance for year-round use, as it attracts healthcare staff to venture outside even during the colder months when the garden itself is dormant. Furthermore, the combination of expansive views and urban feel, with the calmness, safety and privacy of the garden, as well as the microclimate that seemed to enhance the seasons and the expression of nature, is considered difficult to achieve in a ground-level urban garden. This points to the rooftop garden being an urban oasis in the sky with unique, positive and beneficial qualities.

To make the study results easily accessible and useful for professionals working with design and planning of outdoor environments in healthcare, tables (Supplementary Appendix A) clarify the relationship between important garden aspects (in relation to use and experiences), specific physical features and their location in the garden, which indicates, for example, that: A pavilion, preferably heated and with good acoustics, increases use. An attractive view of the city, with easily accessible viewpoints, is a motivating factor for year-round garden visits. A looped path around the garden, wide enough for two people to walk side by side, enables walk n’ talks between colleges. Additional tables in Supplementary Appendix A furthermore show a connection between the above-mentioned garden aspects and the meaning of the roof garden, i.e., how use and experience influence the significance of the garden in relation to well-being and work life sustainability for staff. Examples of these connections are: That looking out over the garden from inside the clinic (use) and experiencing a closeness and connection to nature, weather and seasons (experience), enabling positive distractions (meaningful especially in connection with stressful situations) or That using the garden for short “micro” breaks (use), in an environment that is experienced as aesthetically pleasing (experience), can lead to a feeling of renewed energy for staff (meaningful for health and well-being).

## Data Availability

The raw data supporting the conclusions of this article will be made available by the authors, without undue reservation.
