# Supplementary material for: The roles of an urban rooftop garden for the staff of a memory clinic - a qualitative post-occupancy evaluation
Source: Front Psychol. 2025 Oct 27;16:1646052. doi: 10.3389/fpsyg.2025.1646052 (PMC12599331; doi:10.3389/fpsyg.2025.1646052)
Supplement: Supplementary file 1 [file Data_Sheet_1.pdf]

Appedix A

Table 1. Theme 1a. *Spontaneous visits* (part of The Garden as a place to Use)

**1. The rooftop garden as a place to Use**			
**1a. SPONTANEOUS VISIT**		**Features in the environment**<br>*+ features supporting the use*<br>*- features not supporting the use*	**Place (zone)**
Short "micro" breaks:			
	Taking a walk	+ Looped path around the garden<br>(- drainage problem in one spot)	The garden (zone 3)
	Getting daylight & fresh air		The garden (zone 3)
	Looking at the vegetation, noticing changes	+ Raised planting beds<br>+ Accessible & lush vegetation<br>+ Variety & species richness	The garden (zone 3)
	Looking out over the city	+ Viewpoints in garden<br>+ Unobstructed views<br>+ Safety measures: high railings and fine mesh	The garden and the surrounding (zone 3-4)
	Quick workout	+ Pergola	The garden (zone 3)
Passing trough		+ Paths leading to meeting points (bringing staff through the garden)	Adjacent conference room (zone 1) with doors out to garden.<br>Pavilion (zone 2) located in the garden

Appedix A

Table 2. Theme 1b. *Organized Activity* (part of The Garden as a place to Use)

1. The rooftop garden as a place to Use			
**1b. ORGANIZED ACTIVITY**		**Features in the environment**<br>*+ features supporting the use*<br>*- features not supporting the use*	**Place (zone)**
Longer breaks:			
	Lunch/ coffee	+ Open seating areas<br>+ More private seating areas<br>+ Movable, flexible outdoor furniture<br>+ Areas with choice of sun or shade	The pavilion (zone 2)<br>The garden (the lawn, wooden decked area, smaller garden rooms) (zone 3)
	Enjoying warm and sunny weather	+ Open areas<br>+ Shaded areas	The garden (e.g. the lawn) (zone 3)
Work-related tasks:			
	Staff meetings	<u>The pavilion:</u><br>+ Weather protected<br>+ Technically equipped for meetings and presentations<br>+ Large enough for a group<br>+ Bookable<br><br>- Sometimes cold indoor temperature<br>- Not ideal acoustics<br>- Lack of sun protection for higher windows, causing glare<br>- Loud / noisy ventilation<br><br><u>The garden:</u><br>+ Open seating areas for larger groups<br>+ Smaller seating areas for smaller/ more private meetings<br>+ Movable tables and chairs<br><br>- Level of privacy (confidentiality difficult to ensure)	The pavilion (zone 2)<br>The garden (zone 3)
	Individual work (laptop, reading etc.)	+ A variation of places to sit, with different qualities<br>+ Movable and flexible outdoor furniture<br><br>- Possibility to see laptop screens	The pavilion (zone 2)<br>The garden (zone3)

		(due to sunshine and glare)<br>- Level of privacy (confidentiality difficult to ensure)	
	"Walk & talk" (conversations between colleagues)	+ Looped path, wide enough for two people to walk side by side	The garden (zone 3)
Exercise/ preventive healthcare (walking, mindfulness, gym equipment-work)		+ Looped path<br>+ (Outdoor) Gym equipment<br>+ Open and spacious grass area<br><br>- No privacy for exercise/mindfulness<br>- Exercise equipment a bit difficult to access	The garden (zone 3)
Place to Gather/ Joint activities (kick-offs, celebrations etc.)		+ Open areas, big spaces<br>+ Privacy from people passing by the clinic at ground level<br>+ Connection to the garden from conference room (big windows and glass doors), when too cold to gather outside<br>+ Outdoor lights in garden<br>+ Weather protection	The garden (zone 3)<br>The conference room (zone 2)

Appedix A

Table 3. Theme 2a. *Contact with Nature & Surrounding Life* (part of The garden as a place to Experience the World Outside)

**2. The rooftop garden as a place to Experience the World Outside**		
**2a. CONTACT WITH NATURE & SURROUNDING LIFE**	**Features in the environment**<br>*+ features supporting the connection*<br>*- features not supporting the connection*	**Place (zone)**
Connection and closeness to nature	+ Visual connection through windows<br>+ Closeness to vegetation (e.g. plantings in raised beds, rich vegetation).<br>+ Visiting animals (e.g. birds)<br>+ Views out toward the green part of city<br>+ Fresh air<br>+ Daylight & sunshine	The clinic corridor & conference room (zone 1)<br>The pavilion (zone 2)<br>The garden (zone 3)<br>The city (zone 4)
Experiencing weather and seasons.	+ Visual connection through windows<br>+ Access to the outdoors<br>+ Views out toward the city	The clinic corridor & conference room (zone 1)<br>The pavilion (zone 2)<br>The garden (zone 3)<br>The city (zone 4)
Sensory experiences of nature	+ Fresh air<br>+ Daylight & sunshine<br>+ Variety of plants and flowers (Species richness with different scents, colours, shapes, structures)<br>+ Rippling water feature (- requires care and attention, and sensitive to wind)<br>+ Ground materials with "feeling" (tactile experiences)	The clinic corridor & conference room (zone 1)<br>The pavilion (zone 2)<br>The garden (zone 3)
Openness and views	+ Viewpoints (for access to the edge of the garden)<br>+ Large openings between the pillars in the garden's outer walls<br>+ Fine mesh between the pillars (a safety aspect that does not obstruct the view)<br>+ Glass facades on pavilion & conference room<br>+ Open garden design with no large structure blocking the view<br>+ Attractive views towards the city	The garden (zone 3)<br>The city (zone 4)

	- Possible negative impact on the level of safety and the desired feeling of being enclosed	
Urban oasis in the sky	+ Central location, bustling city<br>+ High up<br>+ Quiet<br>+ Private<br>+ Contrast between incredible proximity to nature & to an urban environment<br>+ Uniqueness (the feeling of being in a special and unique place)<br>+ Impressions (from the world outside)	The garden (zone 3)<br>The city (zone 4)
Easy access	+ Quick & easy to go outside without changing clothes (enables short outdoor breaks)<br>+ Visual contact through windows reminds staff of the garden's existence and encourages use<br>+ Proximity encourages spontaneous use<br><br>- Perceived accessibility varies depending on workplace location (i.e. ease of access experienced unevenly between staff)	From Zone 1 (the clinic) to Zone 3 (the garden)
Enclosure (but not confinement)	+ Sense of safety<br><br>- Possible negative impact on the degree of openness and extent of views from the garden	The garden (zone 3)

Table 4. Theme 2b. *Beyond Hospital Walls* (part of The garden as a place to experience the World Outside)

2. The rooftop garden as a place to Experience the World Outside		
**2b. BEYOND HOSPITAL WALLS** 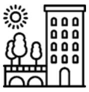	**Features in the environment**<br>*+ features supporting the connection*<br>*- features not supporting the connection*	**Place (zone)**
Aesthetically pleasing design	+ inviting design<br>+ a coherent whole<br>+ beautiful<br>+ plants and flowers<br>+ water feature	The garden (zone 3)
Calmness	+ quiet, calm & peaceful<br>+ low city sounds in background<br>+ calm corners in garden rooms<br>+ no disturbing traffic noise	The garden (zone 3)
Privacy and seclusion	+ variety of seating areas, incl. secluded & private spaces<br>+ smaller garden rooms<br>+ lush and surrounding vegetation<br>+ Being outdoors and still by yourself (instead of out in the city with other people)<br>+ away from the clinic	The pavilion (zone 2)<br>The garden (zone 3)
Social flexibility	+ free, flexible & varying seating options<br>+ fixed, as well as movable furniture	The garden (zone 3)

Appedix A

Table 5. Theme 3a. *Positive & rewarding* (part of The garden as a place of Meaning for Well-being and Work Life Sustainability)

**3. The rooftop garden as a place of Meaning for Well-Being & Work Life Sustainability**		
**Influential aspects** *(Influence of use & experience on the meaning of the garden)*		**3a. POSITIVE & REWARDING** (meaning & significance)
**Theme 1a-b** (Using garden)	**Theme 2 a-b** (Experiencing the garden)	
• Short "micro" breaks<br>• Getting fresh air, daylight, sunshine<br>• Looking out over the city<br>• Walks<br>• Exercise	• Aesthetically pleasing design	***Renewed energy***
• Getting fresh air, daylight, sunshine	• Connection and closeness to nature (& surrounding life)	***Job satisfaction*** *(incl. health promotion & stress preventing)*
• Short "micro" breaks	• Calmness<br>• Easy access<br>• Retreat	***Sense of Freedom***
• Short "micro" breaks<br>• Getting fresh air, daylight, sunshine<br>• Looking out over the city	• View<br>• Aesthetically pleasing design<br>• Retreat<br>• Easy access	***A place of Retreat for the mind***
• Longer breaks (lunch/coffee)<br>• Enjoying sunny weather	• Easy access<br>• Aesthetically pleasing design<br>• Urban oasis in the sky (uniqueness)<br>• Connection & closeness to nature	***Source of Pride*** *(sense of privilege)*

Table 6. Theme 3b. *Temporary wishes & needs for support* (part of The garden as a place of Meaning for Well-being and Work Life Sustainability)

**3. The rooftop garden as a place of Meaning for Well-Being & Work Life Sustainability**		
**Influential aspects** *(Influence of use & experience on the meaning of the garden)*		**3b. TEMPORARY WISHES & NEEDS FOR SUPPORT** (meaning & significance) 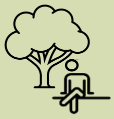
**Theme 1a-b** (Using the garden)	**Theme 2 a-b** (Experiencing the "outside world")	
• Looking out at the garden from inside the clinic	• Connection & closeness to nature<br>• Experiencing weather & seasons<br>• Openness & View	***Positive distractions*** *(view out to garden)*
• Short "micro" breaks<br>• Look at the vegetation, note changes<br>• Look out over the city	• Connection & closeness to nature<br>• Experiencing weather & seasons<br>• Quick & easy access<br>• Openness & view	***Sense of Normality & Hope***
• Exercise (see quote about exercise app in 1b's descriptive text above)<br>• Short & longer breaks	• Refuge<br>• Beyond hospital walls ("a different environment")<br>• Calmness	***Restoration*** *(lower stress levels)*
• Short & longer breaks<br>• Fresh air	• Refuge<br>• Beyond hospital walls ("get away")<br>• Calmness<br>• Openness	***A place to breathe***
